# SARS-CoV-2 Disease Severity and Cycle Threshold Values in Children Infected during Pre-Delta, Delta, and Omicron Periods, Colorado, USA, 2021–2022

**DOI:** 10.3201/eid3006.231427

**Published:** 2024-06

**Authors:** Laura Bankers, Shannon C. O’Brien, Diana M. Tapay, Erin Ho, Isaac Armistead, Alexis Burakoff, Samuel R. Dominguez, Shannon R. Matzinger

**Affiliations:** Colorado Department of Public Health and Environment, Denver, Colorado, USA (L. Bankers, S.C. O’Brien, D.M. Tapay, I. Armistead, A. Burakoff, S.R. Matzinger);; University of Colorado, Aurora, Colorado, USA (E. Ho, S.R. Dominguez);; Children’s Hospital Colorado, Aurora (E. Ho, S.R. Dominguez)

**Keywords:** COVID-19, coronavirus disease, SARS-CoV-2, severe acute respiratory syndrome coronavirus 2, viruses, respiratory infections, zoonoses, COVID-19 pandemic, COVID-19 testing, Ct values, children, whole-genome sequencing, Colorado, United States, Delta, Omicron

## Abstract

In adults, viral load and disease severity can differ by SARS-CoV-2 variant, patterns less understood in children. We evaluated symptomatology, cycle threshold (Ct) values, and SARS-CoV-2 variants among 2,299 pediatric SARS-CoV-2 patients (0–21 years of age) in Colorado, USA, to determine whether children infected with Delta or Omicron had different symptom severity or Ct values than during earlier variants. Children infected during the Delta and Omicron periods had lower Ct values than those infected during pre-Delta, and children <1 year of age had lower Ct values than older children. Hospitalized symptomatic children had lower Ct values than asymptomatic patients. Compared with pre-Delta, more children infected during Delta and Omicron were symptomatic (75.4% pre-Delta, 95.3% Delta, 99.5% Omicron), admitted to intensive care (18.8% pre-Delta, 39.5% Delta, 22.9% Omicron), or received oxygen support (42.0% pre-Delta, 66.3% Delta, 62.3% Omicron). Our data reinforce the need to include children, especially younger children, in pathogen surveillance efforts.

SARS-CoV-2 is characterized by diverse variants (*1*) with differing transmissibility and disease severity (*2*). The rapid evolution and spread of new variants has required a nimble public health response to understand dynamics and clinical implications (*3*), but most work thus far has focused on adults.

The highly transmissible SARS-CoV-2 Delta variant (B.1.617.2) was detected and became the predominant variant in the United States over an 8-week period during May–June 2021 (*4*,*5*). Adults infected by Delta exhibited higher viral loads (*6*) and potentially increased disease severity than those infected by previous variants (*3*,*5*). Taylor et al. (*5*) found a significantly higher proportion of hospitalized patients after Delta became predominant than in earlier pandemic phases. However, in-hospital outcomes did not differ between Delta and pre-Delta variants. Similarly, Twohig et al. (*3*) observed higher risk for hospitalization and emergency care for those infected by Delta than by Alpha (B.1.1.7).

During December 2021, Delta was swiftly overtaken by the more transmissible Omicron (B.1.1.529), which became predominant in the United States over a 2-week period (*4*). Adults infected by Omicron tended to exhibit similar viral loads but lower disease severity compared with Delta-infected adults (*7*). Ulloa et al. (*7*) found reduced risk for hospitalization, intensive care unit (ICU) admission, and death among persons infected by Omicron compared with Delta. Taylor et al. (*8*) observed smaller proportions of hospitalized patients admitted to the ICU or requiring invasive mechanical ventilation and lower rates of in-hospital death with Omicron than Delta. In addition, some studies found that vaccine effectiveness against hospitalization and visits to emergency department or urgent care was lower against Omicron than against Delta (*9*).

Less is known about symptom severity among pediatric patients infected with different variants. Some work suggests little or no difference in disease severity among children infected by Delta compared with previous variants (*10*). However, much of that work has been limited to hospitalized patients. In addition, although the incidence rate of detected infections with Omicron was higher for young children compared with Delta, some studies showed that clinical outcomes of infections with Omicron tended to be less severe than Delta (*11*). However, the large increase in the number of infections during Omicron’s predominance might increase the number of severe outcomes (*12*).

The relationship between cycle threshold (Ct) values and quantitative viral load is tightly inversely correlated (*13*), such that relatively lower Ct values are indicative of higher viral loads. Similar to adults, children who have symptomatic SARS-CoV-2 infections might have lower Ct values (suggesting higher viral loads) compared with those who have asymptomatic infections (*14*), and those infected with Delta or Omicron might have lower Ct values than those infected with other variants (*15*). However, the interplay between SARS-CoV-2 variant, Ct, and symptom severity has not been well studied among pediatric cases, especially among nonhospitalized children.

We evaluated hospitalized and nonhospitalized SARS-CoV-2 pediatric cases in Colorado, USA, during January 2021–January 2022. We used clinical surveillance data, Ct values, and whole-genome sequencing (WGS) to determine whether children infected with either Delta or early Omicron variants had different symptom severity or Ct values from children infected with earlier SARS-CoV-2 variants.

## Methods

### Ethics Statement

The data used in this study were generated for public health surveillance purposes. This activity was determined to be consistent with enhanced disease surveillance activities, not human subjects research, by the Colorado Department of Public Health and Environment’s (CDPHE) Communicable Disease branch. Institutional review board approval was provided by the Colorado Multiple Institutional Review Board. Informed consent was waived.

### Study Population

The study population consisted of hospitalized (hereafter inpatient) and nonhospitalized (hereafter outpatient) cases from the Children’s Hospital Colorado (CHCO) hospitals and outpatient clinics who were Colorado residents <21 years of age and tested positive for SARS-CoV-2 by PCR during January 1, 2021–January 31, 2022. We collected demographic and clinical information including age, race/ethnicity, symptomatology, number of days between symptom onset and positive test (hereafter symptom onset date), and vaccination status. Data were extracted from a state communicable disease database (Colorado Electronic Disease Reporting System), in which communicable diseases are entered as part of public health surveillance and investigation activities.

We performed inpatient chart abstraction to collect data about comorbidities, admission and discharge dates, whether patients were admitted to the hospital because of COVID-19 as opposed to with COVID-19, whether patients were symptomatic because of COVID-19, whether they were admitted to the pediatric ICU (PICU) because of COVID-19, whether they received oxygen support (and type of support) because of COVID-19, and vaccination status. We limited comparisons among inpatients and between inpatients and outpatients to those hospitalized because of COVID-19. We classified patients as admitted because of COVID-19 if their primary complaint symptoms at time of admission to CHCO were consistent with COVID-19 and a COVID-19 test was positive at admission, or if symptoms consistent with COVID-19 developed during hospitalization (with positive test upon admission or later during hospitalization) that would have resulted in admission if they were not already hospitalized. We classified patients as admitted with COVID-19 if they sought care at CHCO with a non–COVID-19 primary diagnosis (e.g., trauma, psychiatry, social reasons, surgery, non–COVID-19 medical diagnoses) and tested positive upon admission on routine surveillance testing. Those persons might also have had >1 symptoms consistent with COVID-19 that did not require admission solely for those symptoms. We determined classification by reviewing clinical provider documentation of assessments and medical decision-making, because International Classification of Diseases, 10th Revision, codes were not consistently available. We considered all common symptoms of COVID-19, including fever, congestion/rhinorrhea, cough, shortness of breath, vomiting/diarrhea, fatigue, headache, and loss of sense of taste/smell.

### RNA Extraction and Quantitative Reverse Transcription PCR

SARS-CoV-2–positive nasopharyngeal swab specimens collected at CHCO hospitals (inpatient) and outpatient clinics (outpatients) were sent to the CDPHE Laboratory. We extracted RNA using the Applied Biosystems MagMAX Viral/Pathogen II Nucleic Acid Isolation Kit on the KingFisher Flex System for automated extraction (ThermoFisher Scientific, https://www.thermofisher.com). We used the Applied Biosystems TaqPath COVID-19 Combo Kit multiplexed reverse transcription PCR (RT-PCR) (ThermoFisher Scientific) to obtain Ct values for open reading frame 1ab (ORF1ab), spike (S), and nucleocapsid (N) gene targets. We used N gene Ct values as a correlate of viral load (*13*).

### Library Preparation and WGS

We performed WGS on either GridION (Oxford Nanopore Technologies, https://www.nanoporetech.com) or NextSeq550 (Illumina, https://www.illumina.com) on all samples that met sequencing criteria (N gene Ct <28). For samples sequenced on GridION, we performed library preparation following the ARTIC tiled PCR amplicon sequencing protocol (*16*,*17*), and for samples sequenced on the NextSeq550, we performed library preparation and single-end Illumina sequencing following the Illumina COVID-Seq assay (*18*) (Appendix).

### Bioinformatic Analysis of WGS Data

We performed assembly and analysis of WGS data on the terra.bio platform (*19*), using our custom, publicly available workflows for SARS-CoV-2 (*20*,*21*) (Appendix). Sequenced samples with >50% coverage are publicly available in the GISAID (https://www.gisaid.org) database and the National Center for Biotechnology Information Sequence Read Archive (BioProject accession no. PRJNA686984) (Appendix Table 1).

### Data Analysis and Statistics

We merged WGS results and deidentified patient data using internal identifiers in Tableau version 2021.4 (*22*). To include data for samples that were not successfully sequenced, we performed analyses in 2 ways.

**Figure 1:**
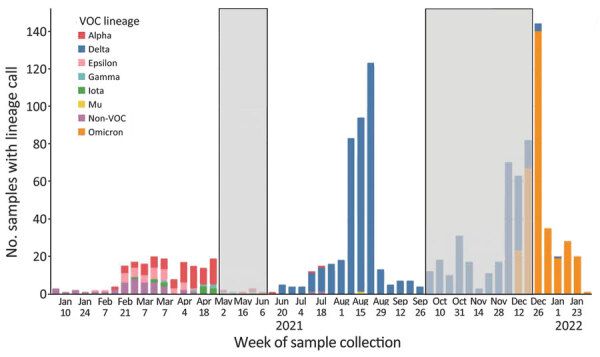
Variant counts of sequencing-confirmed lineages by week of sample collection in study of SARS-CoV-2 disease severity in children during pre-Delta, Delta, and Omicron periods, Colorado, USA, January 2021–January 2022. Gray boxes indicate time periods of potentially mixed lineage that were excluded from time period analyses.

Our first approach mitigates the risk that Ct value analyses could be biased toward lower Ct values because of ability to sequence. We generated a lineage distribution over the course of the study and assigned all samples with Ct values to variant periods. We defined 3 periods on the basis of when Delta or Omicron were at a prevalence of >80% among sequenced Colorado pediatric samples: pre-Delta during January 1–May 1, 2021; Delta during June 15–September 29, 2021; and Omicron during December 26, 2021–January 31, 2022. We excluded samples collected between the pre-Delta and Delta periods and between the Delta and Omicron periods (in which variants were mixed) to improve the accuracy of variant period assignments (Figure 1). Second, to evaluate sensitivity, we performed analyses only on samples that were successfully sequenced and assigned a lineage.

We visualized analyses of Ct values, variant period and lineage, and patient data using Tableau. We performed statistical analyses (analysis of covariance, 1- or 2-way analysis of variance with Tukey test, or χ^2^ test, as appropriate) in Rstudio base packages version 1.4.1106 (RStudio, http://www.rstudio.com). We calculated descriptive characteristics of inpatients including counts and proportions with 95% CI using R version 4.1.1 (The R Foundation for Statistical Computing, https://www.r-project.org). We considered a p value <0.05 statistically significant.

## Results

### Characteristics of Patient Population

The study population included 2,299 persons, 1,629 (70.9%) outpatients and 670 inpatients (29.1%). Of the 670 inpatients, 395 were hospitalized because of COVID-19 and 275 were hospitalized with COVID-19) (Table 1; Appendix Tables 2, 3). Among the entire population, 40.1% were female, 32.6% were non-Hispanic White, 24.4% were Hispanic, and the median age was 6.5 years. A total of 1,724 cases occurred during the pre-Delta (n = 429), Delta (n = 752), and Omicron (n = 543) periods. Proportions of male versus female patients and those with >1 known comorbidity did not differ by variant period (p = 0.44 by χ^2^ test for sex and p = 0.62 by χ^2^ test for comorbidities). However, proportions of racial/ethnic groups differed significantly across variant periods (p<0.0001 by χ^2^ test). 

**Table 1:** Descriptive characteristics of 2,299 children with positive SARS-CoV-2 tests during pre-Delta, Delta, and Omicron periods, Colorado, USA, January 2021–January 2022*

Characteristic	Total	Pre-Delta period	Delta period	Omicron period	p value†
Total population	2,299	429 (18.7, 17.1–20.3)	752 (32.7, 30.8–34.7)	543 (23.6, 21.9–25.4)	
Sex					
F	923 (40.1, 38.1–42.2)	215 (50.1, 45.3–55.0)	352 (46.8, 43.2–50.5)	185 (34.1, 30.1–38.2)	0.44
M	945 (41.1, 39.1–43.2)	198 (46.2, 41.4–51.0)	373 (49.6, 46.0–53.2)	200 (36.8, 32.8–41.0)	
Median age, y (range)	6.5 (0–21)	6.5 (0–20.2)	6.0 (0–21)	6.3 (0–20.9)	
Racial/ethnic group					
Hispanic	560 (24.4, 22.6–26.2)	131 (30.5, 26.2–35.1)	212 (28.2, 25.0–31.6)	127 (23.4, 19.9–27.2)	
Non-Hispanic White	750 (32.6, 30.7–34.6)	203 (47.3, 42.5–52.2)	307 (40.8, 37.3–44.4)	134 (24.7, 21.1–28.5)	<0.0001
Non-Hispanic Black	135 (5.9, 5.0–6.9)	19 (4.4, 2.7–6.8)	58 (7.7, 5.9–9.9)	32 (5.9, 4.1–8.2)	
Non-Hispanic Asian	43 (1.9, 1.4–2.5)	9 (2.1, 1.0–3.9)	17 (2.3, 1.3–3.6)	10 (1.8, 0.9–3.4)	
Unknown‡	673 (29.3, 27.4–31.2)	40 (9.3, 6.7–12.5)	97 (12.9, 10.6–15.5)	213 (39.2, 35.1–43.5)	
Hospitalization status					
Outpatient	1,629 (70.9, 69.0–72.7)	261 (60.8, 56.0–65.5)	619 (82.3, 79.4–85.0)	233 (42.9, 38.7–47.2)	<0.0001
Inpatient	670 (29.1, 27.3–31.1)	168 (39.2, 34.5–44.0)	133 (17.7, 15.0–20.6)	310 (57.1, 52.8–61.3)	
Any comorbidity, inpatient only§	383 (57.2, 53.3–61.0)	101 (60.1, 52.3–67.6)	74 (55.6, 46.8–64.3)	173 (55.8, 50.1–61.4)	0.62
Median time from symptom onset to testing, d (range)	2 (–29 to 320)	2 (–1 to 57)	2 (–2 to 320)	2 (–29 to 44)	
Patients with confirmed sequence	1,177 (51.2, 49.1–53.3)	189 (16.1, 14.0–18.3)	654 (55.6, 52.7–58.4)	334 (28.4, 25.8–31.1)	

*Values are no. (%, 95% CI) except as indicated. p values are indicated as appropriate. †p values were obtained from χ^2^ test of patient characteristics across the 3 variant periods. ‡Unknown racial/ethnic group includes persons with unknown, unknown or not reported, or missing race or ethnicity variables. §Any comorbidity includes history of cardiac, respiratory, gastrointestinal/liver, neurologic, oncologic, obesity, chronic kidney disease, diabetes, or other comorbid condition.

Among inpatients, a larger proportion infected during the Delta or Omicron periods were symptomatic (p<0.0001 by χ^2^ test), admitted to the PICU (p = 0.002 by χ^2^ test), or received oxygen support (p = 0.04 by χ^2^ test) because of COVID-19 compared with persons infected during the pre-Delta period (Table 2; Appendix Tables 4, 5). Mean hospital stay durations were 5.72 (SD +8.30) days for the pre-Delta period, 7.84 (SD +20.6) days for the Delta period, and 3.32 (SD +5.79) days for the Omicron period and were not significantly different (p = 0.1). 

**Table 2:** Number of pediatric COVID-19 inpatients by SARS-CoV-2 variant period and potential indicator of disease severity during pre-Delta, Delta, and Omicron periods, Colorado, USA, January 2021–January 2022*

Indicator of disease severity†	No. (%, 95% CI]				
	Total, n = 395	Pre-Delta, n = 69	Delta, n = 86	Omicron, n = 210	p value‡
Symptomatic	343 (86.8, 83.1–90.0)	52 (75.4, 63.5–84.9)	82 (95.3, 88.5–98.7)	209 (99.5, 97.4–99.9)	<0.0001
Hospitalized	395 (100, 99.1–100)	69 (100, 94.8–100)	86 (100, 95.8–100)	210 (100, 98.3–100)	1.0
PICU admission	94 (23.8, 19.7–28.3)	12 (18.8, 9.3–28.4)	34 (39.5, 29.2–50.7)	48 (22.9, 17.4–29.1)	0.002
Received any oxygen support	217 (54.9, 49.9–59.9)	29 (42.0, 30.2–54.5)	57 (66.3, 55.3–76.1)	131 (62.4, 55.5–69.0)	0.04

*PICU, pediatric intensive care unit. †All indicators of disease severity included here were considered to be a result of COVID-19 illness. ‡p values were obtained from χ^2^ test of patient characteristics across the 3 variant periods.

We obtained Ct values for 1,796 (78.1%) of 2,299 persons; of those, 362 were inpatients and 1,434 were outpatients. Of those 1,796 persons, 1,240 were collected during the 3 variant periods: pre-Delta period (n = 307), Delta period (n = 582), and Omicron period (n = 351). We successfully sequenced 1,276 (55.5%) of 2,299 samples (219 inpatients and 1,057 outpatients) to >50% coverage and obtained lineage calls for 1,177 (51.2%) samples (183 inpatients and 1,053 outpatients; 654 Delta, 334 Omicron, and 189 other).

### Ct Value Patterns across All Variants

**Figure 2:**
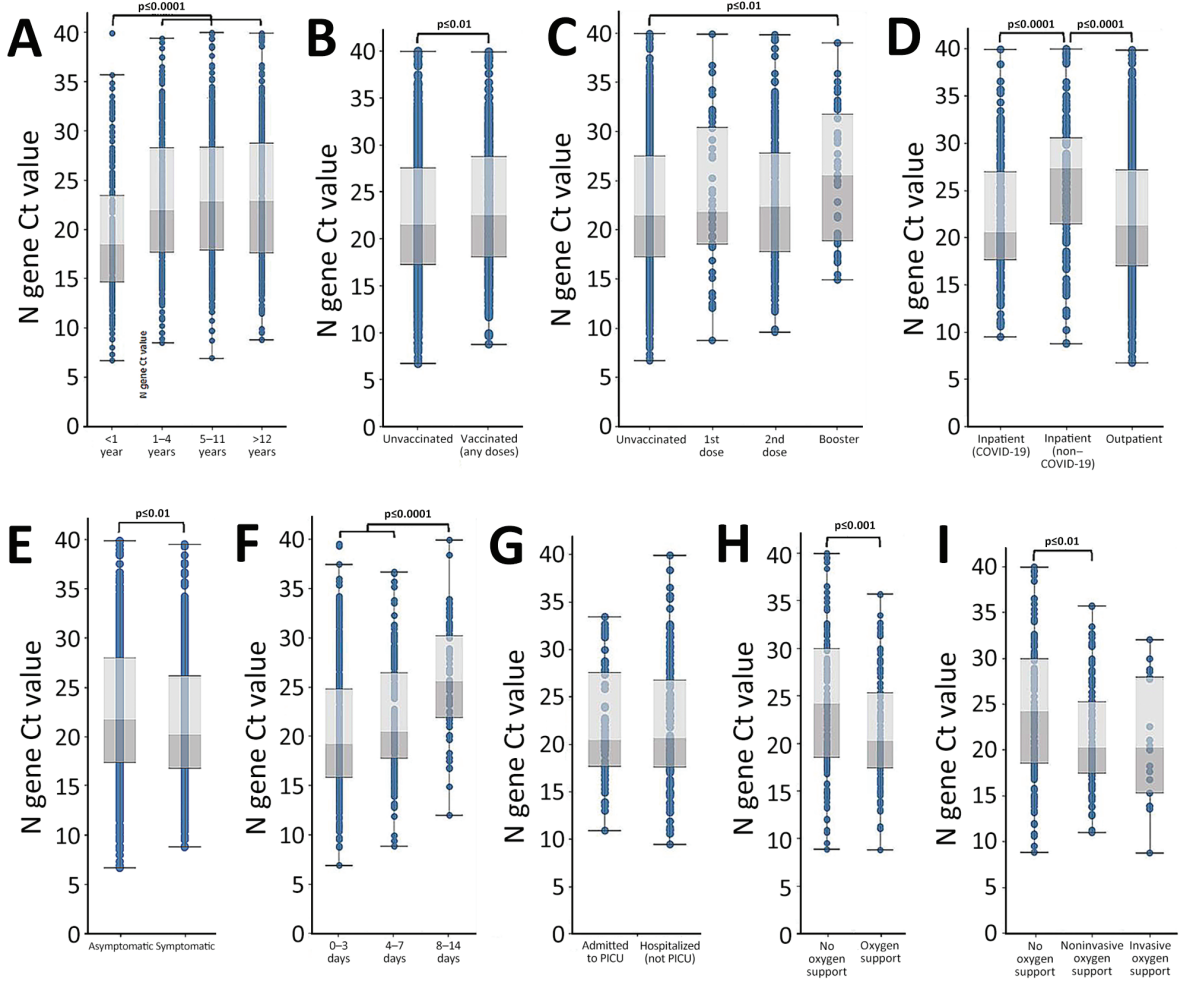
Ct value patterns across all variants in study of SARS-CoV-2 disease severity in children during pre-Delta, Delta, and Omicron periods, Colorado, USA, January 2021–January 2022. Boxplots indicate overall Ct value patterns across categories of patient characteristics, regardless of variant period. A) Age group; B) vaccination status (unvaccinated vs. vaccinated with any number of doses); C) vaccination status (unvaccinated versus vaccinated by number of doses); D) patient type (outpatient/not hospitalized, hospitalized because of COVID-19, hospitalized but not because of COVID-19); E) symptomatic versus asymptomatic; F) number of days between symptom onset and positive test (symptom onset groups); G) hospitalized but not admitted to PICU versus admitted to PICU; H) any type of supplemental oxygen support versus no oxygen support received; I) highest level of supplemental oxygen support received (none, noninvasive oxygen support, invasive oxygen support). Significance was determined using 1-way analysis of variance with Tukey test. Brackets indicate which comparisons correspond to the significance codes, and connected brackets indicate comparisons that have the same significance code. Ct, cycle threshold; PICU, pediatric intensive care unit.

We first evaluated overall patterns of Ct values among our dataset categories. Patients <1 year of age had significantly lower Ct values than the other age groups (adjusted p<0.0001 for 1–4 years, 5–11 years, and >12 years) (Table 3; Figure 2). We observed the same pattern among patients <1 year of age when limited to sequenced samples (adjusted p = 0.0003 for patients 1–4 years of age, adjusted p = 0.0002 for 5–11 years, and adjusted p = 0.0002 for >12 years) (Appendix Tables 6–8, Figure 1). Patients who received any number of vaccine doses had significantly higher Ct values than unvaccinated patients (adjusted p = 0.003). In addition, patients who had received a booster had significantly higher Ct values than unvaccinated patients (adjusted p = 0.0081).

**Table 3:** Mean nucleocapsid gene cycle threshold values by category in study of SARS-CoV-2 disease severity in children during pre-Delta, Delta, and Omicron periods, Colorado, USA, January 2021–January 2022*

Characteristic	Cycle threshold (+SD)			
	All	Pre-Delta period	Delta period	Omicron period
All	22.4 (6.79)	26.5 (6.96)	22.8 (7.81)	22.9 (5.51)
Age group, y				
<1	19.5 (6.54)	22.0 (7.49)	17.2 (6.64)	21.4 (5.79)
1–4	22.6 (6.74)	24.7 (6.53)	21.4 (7.61)	21.9 (4.82)
5–11	23.2 (6.61)	23.1 (7.07)	23.1 (6.85)	23.0 (5.44)
>12	23.3 (6.73)	24.1 (7.28)	22.2 (6.77)	24.7 (5.39)
Time from symptom onset, d				
0–3	20.7 (6.32)	21.5 (6.36)	20.5 (6.95)	20.4 (4.71)
4–7	21.9 (5.85)	20.1 (5.02)	22.1 (6.49)	22.3 (4.47)
8–14	25.8 (5.58)	29.0 (6.22)	24.7 (5.79)	26.7 (3.48)
Vaccination status				
Unvaccinated	22.2 (6.53)	23.6 (7.17)	21.2 (7.31)	22.2 (5.53)
Vaccinated (any doses)	23.2 (6.76)	24.1 (6.99)	22.9 (6.87)	22.8 (4.67)
Partially vaccinated (1 dose)	23.4 (7.45)	28.5 (7.33)	21.0 (6.43)	23.7 (5.16)
Fully vaccinated (2 doses)	23.0 (6.59)	22.5 (6.51)	23.0 (6.91)	22.8 (4.80)
Fully vaccinated + booster	25.3 (6.81)	25.9 (6.92)	24.5 (7.26)	20.7 (3.04)
Disease severity				
Outpatient	22.1 (6.47)	22.4 (6.79)	21.4 (7.07)	21.9 (5.40)
Inpatient, hospitalized with COVID-19	26.2 (6.92)	27.9 (6.93)	24.3 (8.25)	25.5 (5.70)
Inpatient, hospitalized because of COVID-19	22.1 (6.31)	24.6 (6.62)	22.1 (7.53)	21.3 (4.75)
Symptomatic	21.5 (6.37)	21.6 (6.24)	21.7 (7.02)	21.4 (5.24)
Asymptomatic	22.5 (6.80)	23.7 (6.99)	21.6 (7.29)	22.4 (5.43)
Admitted to PICU because of COVID-19	21.9 (5.84)	22.5 (5.95)	22.3 (6.88)	21.0 (4.17)
Hospitalized but not admitted to PICU	22.1 (6.50)	25.3 (6.76)	21.9 (8.04)	21.3 (4.97)
No supplemental oxygen	24.3 (7.16)	27.8 (7.62)	22.3 (8.35)	23.5 (5.12)
Any supplemental oxygen	21.3 (5.82)	22.1 (6.55)	22.1 (6.60)	20.7 (4.79)
Noninvasive supplemental oxygen	21.4 (5.67)	22.2 (5.59)	22.7 (6.66)	20.4 (4.72)
Invasive supplemental oxygen	20.9 (6.82)	22.0 (10.2)	18.7 (5.40)	22.7 (5.35)

*PICU, pediatric intensive care unit.

Regarding potential disease severity indicators, we found that inpatients hospitalized with COVID-19 had significantly higher Ct values than both inpatients hospitalized because of COVID-19 (adjusted p<0.0001) and outpatients (adjusted p<0.0001). Ct values for inpatients hospitalized because of COVID-19 and outpatients did not differ significantly from each other (Table 3; Figure 2). Symptomatic patients had significantly lower Ct values than asymptomatic patients (adjusted p = 0.0081). Those tested soon after symptom onset had lower Ct values than patients tested 8–14 days after symptom onset (adjusted p<0.0001 for 0–3 days and 4–7 days after symptom onset). Similarly, Ct values were significantly lower in the groups tested 0–3 days and 4–7 days after symptom onset than in the 8–14 days group when limited to sequenced samples (adjusted p<0.0001 for 0–3 days, adjusted p = 0.0055 for 4–7 days) (Appendix Tables 6–8, Figure 1). 

Among inpatients, we did not observe a significant difference in Ct values according to PICU admission status. However, those who received any type of supplemental oxygen support (invasive or noninvasive) and those who received noninvasive supplemental oxygen had lower Ct values than those who did not receive any form of supplemental oxygen (adjusted p = 0.0002 for any type of supplemental oxygen and adjusted p = 0.0012 for noninvasive supplemental oxygen).

### Ct Value Patterns by Variant Period

**Figure 3:**
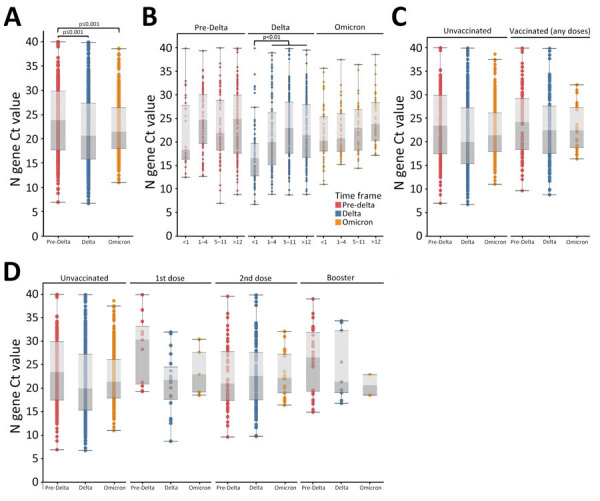
Ct value patterns of patient characteristics across variant periods in study of SARS-CoV-2 disease severity in children during pre-Delta, Delta, and Omicron periods, Colorado, USA, January 2021–January 2022. Boxplots indicate overall Ct value patterns among patient characteristics across variant periods. A) Variant period; B) age group; C) vaccination status (unvaccinated vs. vaccinated with any number of doses); D) vaccination status (unvaccinated vs. vaccinated by number of doses). Red = pre-Delta, Blue = Delta, Orange = Omicron. Significance was determined using 1-way analysis of variance (A) or 2-way (B–D) with Tukey test. Brackets indicate which comparisons correspond to the significance codes, and connected brackets indicate comparisons that have the same significance code. Ct, cycle threshold.

**Figure 4:**
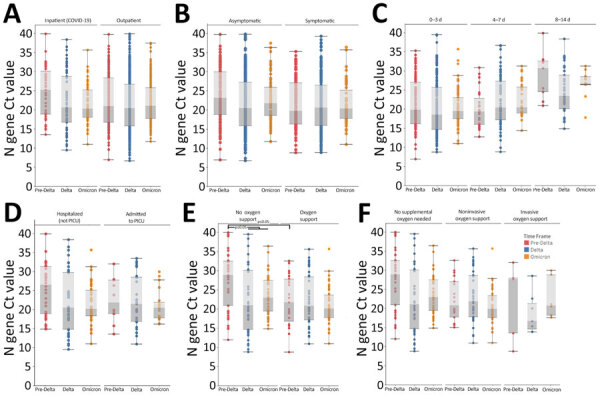
Ct value patterns among markers of disease severity across variant periods in study of SARS-CoV-2 disease severity in children during pre-Delta, Delta, and Omicron periods, Colorado, USA, January 2021–January 2022. Boxplots indicate overall Ct value patterns across disease severity markers by variant period. A) Patient type (outpatient/not hospitalized, hospitalized because of COVID-19); B) symptomatic versus asymptomatic; C) number of days between symptom onset and positive test (symptom onset groups); D) hospitalized but not admitted to PICU versus admitted to PICU; E) any type of supplemental oxygen support versus no oxygen support received; F) highest level of supplemental oxygen support received (none, noninvasive oxygen support, invasive oxygen support). Red = pre-Delta, Blue = Delta, Orange = Omicron. Significance was determined using 2-way analysis of variance with Tukey test. Brackets indicate which comparisons correspond to the significance codes, and connected brackets indicate comparisons that have the same significance code. Ct, cycle threshold; PICU, pediatric intensive care unit.

We analyzed Ct value patterns for each dataset category across variant periods (Table 3; Figures 3, 4) as well as among lineages for sequenced samples (Appendix Tables 6–8, Figures 2 and 3). Ct values were significantly lower during the Delta and Omicron periods than in the pre-Delta period (adjusted p value = 0.0004 for Delta period and adjusted p = 0.0003 for Omicron period) but did not differ significantly between Delta and Omicron (Table 3; Figure 3). We did not observe this pattern in the subset of sequenced samples (Appendix Tables 6–8, Figure 2). We observed a significant interaction between variant period and patient age (adjusted p = 0.001). Among patients infected during the Delta period, children <1 year of age had significantly lower Ct values than those >1 year of age (adjusted p = 0.0021 for children 1–4 years of age, adjusted p<0.0001 for 5–11 years, and adjusted p<0.0001 for >12 years) (Table 3; Figure 3). In addition, patients <1 year of age infected during Delta had significantly lower Ct values than children of the same age group infected during the Omicron period (adjusted p = 0.0156). Patients infected during the Delta period who were 1–4 years of age had lower Ct values than children in the same age group who were infected during the pre-Delta period (adjusted p = 0.031). We did not observe a significant interaction between lineage and patient age among sequenced samples (Appendix Tables 6–8, Figure 2). Furthermore, we did not observe a significant interaction between either variant period or sequenced lineage and vaccination status (Table 3; Figure 3; Appendix Tables 6–8, Figure 2).

When we evaluated potential indicators of disease severity across variant periods, we did not observe a statistically significant interaction between the variant period and patient type (inpatient because of COVID-19 vs. outpatient), the presence or absence of symptoms, days from symptom onset, or whether inpatients were admitted to the PICU (Table 3; Figure 4). Among patients who did not receive supplemental oxygen, those infected during the pre-Delta period had higher Ct values than those infected during the Delta (adjusted p = 0.0017) or Omicron (adjusted p = 0.0238) periods, in addition to having higher Ct values than children infected during pre-Delta who received supplemental oxygen (adjusted p = 0.0121). However, when compared by the highest level of supplemental oxygen support received (none, invasive, or noninvasive), results were not statistically significant. Among sequenced samples, we did not observe any statistically significant interactions between lineage and disease severity indicators, likely because of Ct value bias and smaller sample sizes (Appendix Tables 6–8, Figure 3).

## Discussion

We found that children infected with SARS-CoV-2 during the Delta and Omicron periods had lower Ct values (suggesting higher viral load) than children infected during the pre-Delta period. Patients who were <1 year of age had lower Ct values than children in other age groups, a pattern potentially driven by Delta. In addition, inpatients with symptomatic infections had lower Ct values than asymptomatic inpatients, but we did not observe significant differences in Ct values among disease severity markers among variant periods. Therefore, Ct value might not be a strong correlate of disease severity outcomes across variant periods in children. During the Delta and Omicron periods, a greater proportion of inpatients were symptomatic, hospitalized in the PICU, or received oxygen support because of COVID-19 than during the pre-Delta period, but those proportions did not differ significantly between Delta and Omicron. This finding suggests that, for hospitalized children, Delta and Omicron infections might be more severe than earlier variants, regardless of Ct value.

Although many studies have evaluated relationships between SARS-CoV-2 variants and Ct values or viral load (*6*,*7*), transmission (*23*), and symptom severity (*3*,*5*,*7*) in adults, fewer studies have focused on children. Therefore, the effects of SARS-CoV-2 variant on Ct values or viral load and symptom severity in this population remain poorly understood.

Previous studies in adults observed that those infected by Delta exhibit higher viral loads (*6*) than those infected by previous variants and those infected by Omicron exhibit similar viral loads to those infected by Delta (*7*). Consistent with our results, previous studies in children observed lower Ct values in hospitalized children infected with Delta and Omicron than for previous variants (*15*). On the other hand, studies regarding the relationship between viral load and patient age yielded mixed results (*24*). Consistent with our results, others have found that infants (*25*) and young children (*26*,*27*) exhibit higher viral loads than older children. One study observed that older children exhibit similar viral loads to adults (*25*), whereas others found no significant effect of age on viral load (*28*–*30*). Therefore, more work with larger sample sizes or meta-analyses will be required to describe the relationship between age and Ct values or viral load in pediatric cases.

Although not directly addressed in this study, differences in viral loads among age groups or among SARS-CoV-2 variants have the potential to influence SARS-CoV-2 transmission dynamics. Early household transmission studies found that children transmit SARS-CoV-2 at lower rates than adults (*23*,*31*). However, more recent studies showed increased transmission by children in schools (*32*) and athletic facilities (*33*) during Delta compared with earlier variants. In addition, Omicron is more contagious than previous variants, including Delta (*34*). Finally, Costa et al. (*29*) found faster RNA clearance in children than in adults, suggesting a shorter viral shedding period, which might contribute to differing transmission dynamics between adults and children. This finding suggests that children might have a more significant effect on SARS-CoV-2 transmission dynamics than previously thought.

Consistent with our study, Chung et al. (*28*) found lower Ct values in symptomatic children than asymptomatic children. However, we did not observe a significant relationship between Ct values and markers of disease severity across variant periods among hospitalized children. Instead, higher proportions of hospitalized children exhibited disease severity markers during the Delta and Omicron periods than during pre-Delta, regardless of Ct values. Similarly, Quintero et al. (*15*) found that children infected by Delta were more likely to be symptomatic than those infected by earlier variants, and Mitchell et al. (*35*) found the highest proportion of PICU admissions after the emergence of Omicron. A review by Khemiri et al. (*36*) observed similar clinical manifestations in children and adults infected by Delta and Omicron. Furthermore, children, particularly those <5 years of age, were at higher risk for hospitalization (*8*,*37*–*39*) and had a higher incidence of croup (*40*) during Omicron than during earlier variants. In sum, those studies suggest that Delta and Omicron infections might be more severe in children than previous variants.

The first limitation of our study is that analyses of the relationship between WGS-based SARS-CoV-2 lineage calls and Ct values might be biased toward lower Ct values because the CDPHE Laboratory uses a cutoff of <28 Ct for WGS. As such, we performed our analyses using variant periods to include all samples for which we had Ct data, regardless of whether they were successfully sequenced, with the understanding that a small number of samples might be assigned to the incorrect variant period. We found that our analysis lost some power when only looking at samples that were successfully sequenced, but the results largely agree with the full dataset that used variant periods. Second, although the relationship between Ct values and quantitative viral load are not exact, they are tightly inversely correlated (*13*). Because we used a single quantitative RT-PCR protocol and platform for all samples, we expect the Ct patterns, as they relate to viral load, to be consistent. Third, whether samples with high Ct values represent the beginning or end of infection is unclear. However, it is assumed that most patients are tested early in the course of infection. Fourth, because we concluded data collection in January 2022, our analyses of Omicron only include the sublineages BA.1.1.529, BA.1, and BA.1.1. Because of the rapid evolution of SARS-CoV-2, numerous Omicron sublineages have emerged since and are not included here. Those other sublineages could exhibit different characteristics, such as different Ct values and altered transmissibility or virulence (*34*). Future work could address those differences among Omicron sublineages. Finally, our analyses only included Colorado residents and therefore might not be generalizable to other jurisdictions or geographic locations.

Our study provides insight into the relationship between Ct values, the presence of symptoms, and disease severity markers across variant periods. A strength of our study is the inclusion of in-depth data for both inpatient and outpatient populations. Similar studies are limited to performing in-depth analyses on Ct values to inpatient populations (*37*). In addition, our study is unique in its integration of clinical and laboratory data. Furthermore, rather than relying on RT-PCR of a small number of mutations to delineate variant periods, we were able to use entire genomes, enabling more accurate lineage determination.

In conclusion, our results suggest that children infected with either Delta or Omicron had lower Ct values (suggesting higher viral load) and potentially greater disease severity than children infected with earlier variants. Those patterns were particularly pronounced in the youngest children (<1 year of age). Our data highlight the importance of monitoring children, particularly in younger age groups, as new variants emerge and including pediatric cases in surveillance efforts for current and future SARS-CoV-2 variants to understand the potential differences in Ct values and viral loads, transmission, and disease severity across the age spectrum.

Appendix SARS-CoV-2 disease severity and cycle threshold values in children infected during pre-Delta, Delta, and Omicron periods, Colorado, USA.
