# Supplementary material for: SARS-CoV-2 Disease Severity and Cycle Threshold Values in Children Infected during Pre-Delta, Delta, and Omicron Periods, Colorado, USA, 2021–2022
Source: Emerg Infect Dis. 2024 Jun;30(6):1182–92. doi: 10.3201/eid3006.231427 (PMC11139003; doi:10.3201/eid3006.231427)
Supplement: Appendix — SARS-CoV-2 disease severity and cycle threshold values in children infected during pre-Delta, Delta, and Omicron periods, Colorado, USA. [file 23-1427-Techapp-s1.pdf]

Article DOI: https://doi.org/10.3201/eid3006.231427

EID cannot ensure accessibility for supplementary materials supplied by authors. Readers who have difficulty accessing supplementary content should contact the authors for assistance.

SARS-CoV-2 Disease Severity and Cycle Threshold Values in Children Infected during Pre-Delta, Delta, and Omicron Periods, Colorado, USA, 2021–2022

Appendix

Supplementary Methods

Library preparation and whole genome sequencing

Whole genome sequencing was performed on either an Oxford Nanopore (ONT) GridION instrument (Oxford Nanopore Technologies, Oxford, UK) or an Illumina NextSeq 550 instrument (Illumina, San Diego, CA, USA) on all samples that met sequencing criteria (N gene Ct of 28 or less). For samples sequenced on ONT GridION, library preparation for whole genome sequencing was performed following the ARTIC tiled PCR amplicon sequencing protocol.

For samples that were sequenced on the ONT GridION, an internally optimized version of the ARTIC V3 protocol for Illumina was followed, through the “Amplified cDNA SPRI” section (*1*), and then the ARTIC V3 protocol for ONT beginning at the “native barcoding” section (*2*), followed by sequencing on the ONT GridION.

For samples that were sequenced on the NextSeq 550, library preparation and single-end Illumina sequencing were performed following an internally optimized version of the Illumina COVID-Seq assay (*3*).

Bioinformatic analysis of whole genome sequencing data

All of our bioinformatics workflows were written in WDL (Workflow Development Language), are compatible with Google Cloud and the Broad Institute's terra.bio platform (*4*), and have been made publicly available on github (*5*) and dockstore (*6*). For samples sequenced on ONT GridION, quality filtering and trimming of reads and reference-guided assembly were performed using our nanopore-preprocessing-assembly.wdl workflow. For samples sequenced on Illumina NextSeq, quality filtering and trimming of reads and reference-guided assembly were performed using our COVIDseqSEassembly.wdl workflow. Lineage assignment was performed on all resulting consensus genome assemblies using our classifyCOVIDlineage.wdl, which uses NextClade and Pangolin to assign clade and lineage information. Among sequenced samples, we obtained a mean base quality of 29.4 (SD 6.6), mean number of filtered reads per sample of 392,256.01 (SD 397,572.6), mean sequencing depth of 1013.2 (SD 719.6), mean mapping quality of 59.9 (SD 0.4), and mean percent reference coverage of 94.3 (SD 7.8). All sequenced samples with at least 50% coverage were made publicly available on GISAID and NCBI (Table S1).

References

1. DNA Pipelines R&D, Farr B, Rajan D, Betteridge E, Shirley L, Quail M, et al. COVID-19 ARTIC v3 Illumina library construction and sequencing protocol. 2020 Nov 4 [cited 2022 Feb 15]. https://www.protocols.io/view/covid-19-artic-v3-illumina-library-construction-an-j8nlke665l5r/v5

2. Quick J. nCoV-2019 sequencing protocol v3 (LoCost). 2020 Aug 25 [cited 2022 Feb 15]. https://www.protocols.io/view/ncov-2019-sequencing-protocol-v3-locost-bp2l6n26rgqe/v3

3. Illumina CovidSeq Test Instructions for use [cited 2022 Feb 15]. https://support.illumina.com/content/dam/illumina-support/documents/documentation/chemistry_documentation/Illumina-COVIDSeq-Test/illumina-covidseq-test-instructions-for-use-1000000128490-03.PDF.

4. The Broad Institute. Terra [cited 2022 Feb 22]. https://app.terra.bio/.

5. Colorado Department of Public Health and Environment. CDPHE-bioinformatics/CDPHE-SARS-CoV-2 [cited 2022 Feb 22]. https://github.com/CDPHE-bioinformatics/CDPHE-SARS-CoV-2

6. Colorado Department of Public Health and Environment. CDPHEsarscov2 [cited 2022 Feb 22]. https://dockstore.org/organizations/CDPHE/collections/CDPHEsarscov2.

Appendix Table 1. NCBI and GISAID accession IDs for all samples that were successfully sequenced to at least 50% reference genome coverage and submitted to public repositories

BioProject	BioSample	SRA	GenBank	GISAID
PRJNA686984	SAMN18006872	SRR13748705	MW648322	EPI_ISL_1038922
PRJNA686984	SAMN18174038	SRR13867996	MW715269	EPI_ISL_1169636
PRJNA686984	SAMN18174039	SRR13867995	MW715270	EPI_ISL_1169806
PRJNA686984	SAMN18174040	SRR13867994	MW715271	EPI_ISL_1169622
PRJNA686984	SAMN18174041	SRR13867993	MW715272	EPI_ISL_1169638
PRJNA686984	SAMN18174048	SRR13867985	MW715279	EPI_ISL_1169667
PRJNA686984	SAMN23283405	SRR17019294	OL678666	EPI_ISL_6582052
PRJNA686984	SAMN18290939	SRR13958069	MW796771	EPI_ISL_1234143
PRJNA686984	SAMN18290940	SRR13958068	MW796772	EPI_ISL_1234144
PRJNA686984	SAMN18290941	SRR13958066	MW796773	EPI_ISL_1234145
PRJNA686984	SAMN18290942	SRR13958065	MW796774	EPI_ISL_1234146
PRJNA686984	SAMN18290943	SRR13958064	MW796775	EPI_ISL_1234147
PRJNA686984	SAMN18290946	SRR13958061	MW796778	EPI_ISL_1234150
PRJNA686984	SAMN18290947	SRR13958060	MW796779	EPI_ISL_1234151
PRJNA686984	SAMN18290948	SRR13958059	MW796780	EPI_ISL_1234152
PRJNA686984	SAMN18290949	SRR13958058	MW796781	EPI_ISL_1234153
PRJNA686984	SAMN18290950	SRR13958057	MW796782	EPI_ISL_1234154
PRJNA686984	SAMN18291099	SRR13958247	MW796923	EPI_ISL_1234303
PRJNA686984	SAMN18291155	SRR13958185	MW796979	EPI_ISL_1234359
PRJNA686984	SAMN18291156	SRR13958184	MW796980	EPI_ISL_1234360
PRJNA686984	SAMN18291158	SRR13958182	MW796982	EPI_ISL_1234362
PRJNA686984	SAMN18291159	SRR13958181	MW796983	EPI_ISL_1234363
PRJNA686984	SAMN23283686	SRR17019109	OL678681	EPI_ISL_6582328
PRJNA686984	SAMN18291160	SRR13958180	MW796984	EPI_ISL_1234364
PRJNA686984	SAMN18291161	SRR13958110	MW796985	EPI_ISL_1234365
PRJNA686984	SAMN18291162	SRR13958109	MW796986	EPI_ISL_1234366
PRJNA686984	SAMN18291163	SRR13958108	MW796987	EPI_ISL_1234367
PRJNA686984	SAMN18291164	SRR13958107	MW796988	EPI_ISL_1234368
PRJNA686984	SAMN18291165	SRR13958106	MW796989	EPI_ISL_1234369
PRJNA686984	SAMN18291166	SRR13958105	MW796990	EPI_ISL_1234370
PRJNA686984	SAMN18291167	SRR13958104	MW796991	EPI_ISL_1234371
PRJNA686984	SAMN18291168	SRR13958103	MW796992	EPI_ISL_1234372
PRJNA686984	SAMN18291169	SRR13958102	MW796993	EPI_ISL_1234373
PRJNA686984	SAMN18291172	SRR13958097	MW796996	EPI_ISL_1234376
PRJNA686984	SAMN19408144	SRR17001618	OL891363	EPI_ISL_1539448
PRJNA686984	SAMN19408146	SRR17001990	OL890740	EPI_ISL_1540119
PRJNA686984	SAMN19408151	SRR17001601	OL891445	EPI_ISL_1539672
PRJNA686984	SAMN19408153	SRR17002002	OL891141	EPI_ISL_1539579
PRJNA686984	SAMN19408155	SRR17001997	OL891256	EPI_ISL_1539991
PRJNA686984	SAMN19403994	SRR17001994	OL890710	EPI_ISL_2194662
PRJNA686984	SAMN19408157	SRR17001978	OL890995	EPI_ISL_1539374
PRJNA686984	SAMN19408158	SRR17001627	OL891052	EPI_ISL_1540310
PRJNA686984	SAMN19408596	SRR17350065	OM067980	EPI_ISL_1540049
PRJNA686984	SAMN19408599	SRR17350087	OM067327	EPI_ISL_1540391
PRJNA686984	SAMN19408600	SRR17350676	OM067867	EPI_ISL_1539834
PRJNA686984	SAMN19408602	SRR17350261	OM067905	EPI_ISL_1540051
PRJNA686984	SAMN19408605	SRR17350579	OM067189	EPI_ISL_1540393
PRJNA686984	SAMN19404057	SRR17350288	OM067521	EPI_ISL_2194725
PRJNA686984	SAMN19408606	SRR17350617	OM067949	EPI_ISL_1540394
PRJNA686984	SAMN19408607	SRR17350870	OM067419	EPI_ISL_1539835
PRJNA686984	SAMN19408608	SRR17350391	OM067487	EPI_ISL_1540395
PRJNA686984	SAMN19408611	SRR17350637	OM067161	EPI_ISL_1539536
PRJNA686984	SAMN19408612	SRR17350648	OM067182	EPI_ISL_1539537
PRJNA686984	SAMN19408651	SRR17350938	Not submitted	EPI_ISL_1540104
PRJNA686984	SAMN19408652	SRR17350915	OM067191	EPI_ISL_1540398
PRJNA686984	SAMN19408657	SRR17350667	OM067260	EPI_ISL_1540401
PRJNA686984	SAMN19409164	SRR17350107	OM067418	EPI_ISL_1617040
PRJNA686984	SAMN19409165	SRR17350108	OM067999	EPI_ISL_1617041

BioProject	BioSample	SRA	GenBank	GISAID
PRJNA686984	SAMN19409167	SRR17350110	OM067125	EPI_ISL_1617043
PRJNA686984	SAMN19404116	SRR17350621	OM067988	EPI_ISL_2194784
PRJNA686984	SAMN19404117	SRR17350627	OM067430	EPI_ISL_2194785
PRJNA686984	SAMN19404118	SRR17350276	OM067899	EPI_ISL_2194786
PRJNA686984	SAMN19404119	SRR17350278	OM067706	EPI_ISL_2194787
PRJNA686984	SAMN19404120	SRR17350114	OM067244	EPI_ISL_2194788
PRJNA686984	SAMN19409176	SRR17350296	OM067777	EPI_ISL_1617052
PRJNA686984	SAMN19409331	SRR17324387	OM068275	EPI_ISL_1617210
PRJNA686984	SAMN19409332	SRR17324377	OM068236	EPI_ISL_1617211
PRJNA686984	SAMN19404221	SRR17324088	OM068302	EPI_ISL_2194889
PRJNA686984	SAMN19409337	SRR17324393	OM068285	EPI_ISL_1617217
PRJNA686984	SAMN19404224	SRR17324087	OM068526	EPI_ISL_2194892
PRJNA686984	SAMN19404228	SRR17323686	OM068222	EPI_ISL_2194896
PRJNA686984	SAMN19404374	SRR17324270	OM068380	EPI_ISL_2195042
PRJNA686984	SAMN19409617	SRR17323836	OM068594	EPI_ISL_1665455
PRJNA686984	SAMN19409620	SRR17324410	OM068123	EPI_ISL_1665458
PRJNA686984	SAMN19404528	SRR17415692	OM163417	EPI_ISL_2195196
PRJNA686984	SAMN19409790	SRR17416144	OM163853	EPI_ISL_1665629
PRJNA686984	SAMN19404697	SRR17416617	OM163294	EPI_ISL_2195364
PRJNA686984	SAMN19404698	SRR17416619	OM163325	EPI_ISL_2195365
PRJNA686984	SAMN19408921	SRR17416637	Not submitted	EPI_ISL_1665894
PRJNA686984	SAMN19404699	SRR17416620	OM163206	EPI_ISL_2195366
PRJNA686984	SAMN19404700	SRR17416621	OM163648	EPI_ISL_2195367
PRJNA686984	SAMN19408922	SRR17416638	OM163089	EPI_ISL_1665895
PRJNA686984	SAMN19404701	SRR17416622	OM163094	EPI_ISL_2195368
PRJNA686984	SAMN19408923	SRR17416639	OM163077	EPI_ISL_1665896
PRJNA686984	SAMN19404703	SRR17416624	OM163085	EPI_ISL_2195370
PRJNA686984	SAMN19404704	SRR17416625	OM163204	EPI_ISL_2195371
PRJNA686984	SAMN19404705	SRR17416626	OM163506	EPI_ISL_2195372
PRJNA686984	SAMN19410190	SRR17416599	OM163530	EPI_ISL_2276297
PRJNA686984	SAMN19410191	SRR17416600	OM163271	EPI_ISL_2276298
PRJNA686984	SAMN19408924	SRR17416641	OM163442	EPI_ISL_1665897
PRJNA686984	SAMN19410523	SRR17416041	OM163716	EPI_ISL_2276630
PRJNA686984	SAMN19410525	SRR17416039	OM163185	EPI_ISL_2276632
PRJNA686984	SAMN19410526	SRR17416020	OM163471	EPI_ISL_2276633
PRJNA686984	SAMN19410527	SRR17416022	OM164005	EPI_ISL_2276634
PRJNA686984	SAMN19410533	SRR17416029	OM163443	EPI_ISL_2276640
PRJNA686984	SAMN19411078	SRR17416998	OM138146	EPI_ISL_2277183
PRJNA686984	SAMN19411081	SRR17416997	Not submitted	EPI_ISL_2277186
PRJNA686984	SAMN19411082	SRR17417000	OM137983	EPI_ISL_2277187
PRJNA686984	SAMN19411083	SRR17417001	OM138308	EPI_ISL_2277188
PRJNA686984	SAMN23071206	SRR16920640	OL544657	EPI_ISL_6211092
PRJNA686984	SAMN23071207	SRR16920639	OL544612	EPI_ISL_6211093
PRJNA686984	SAMN23071208	SRR16920214	OL544196	EPI_ISL_6211094
PRJNA686984	SAMN23071209	SRR16920213	OL544621	EPI_ISL_6261148
PRJNA686984	SAMN23071210	SRR16920212	OL544398	EPI_ISL_6211095
PRJNA686984	SAMN23071211	SRR16920211	OL544327	EPI_ISL_6211096
PRJNA686984	SAMN23071212	SRR16920210	OL544614	EPI_ISL_6211097
PRJNA686984	SAMN23071213	SRR16920209	OL544622	EPI_ISL_6211098
PRJNA686984	SAMN19411499	SRR17417373	OM138272	EPI_ISL_2277603
PRJNA686984	SAMN19411501	SRR17417376	OM138618	EPI_ISL_2277605
PRJNA686984	SAMN19411502	SRR17417377	OM138407	EPI_ISL_2277606
PRJNA686984	SAMN19411503	SRR17417378	OM138082	EPI_ISL_2277607
PRJNA686984	SAMN19411504	SRR17417379	OM138077	EPI_ISL_2277608
PRJNA686984	SAMN19411505	SRR17417380	OM138683	EPI_ISL_2277609
PRJNA686984	SAMN19411694	SRR17435360	OM249133	EPI_ISL_2309117
PRJNA686984	SAMN19411695	SRR17435361	OM249291	EPI_ISL_2309118
PRJNA686984	SAMN19411696	SRR17435362	OM248815	EPI_ISL_2309119
PRJNA686984	SAMN19411697	SRR17435363	OM249284	EPI_ISL_2309120
PRJNA686984	SAMN19411699	SRR17435365	OM249429	EPI_ISL_2309122
PRJNA686984	SAMN19411701	SRR17435368	OM249249	EPI_ISL_2309124
PRJNA686984	SAMN19411702	SRR17436095	OM249383	EPI_ISL_2309125
PRJNA686984	SAMN19411704	SRR17435370	OM248733	EPI_ISL_2309127
PRJNA686984	SAMN19412134	SRR17435741	OM249593	EPI_ISL_2309557
PRJNA686984	SAMN19412143	SRR17435728	OM248727	EPI_ISL_2309566
PRJNA686984	SAMN19412144	SRR17435720	OM248757	EPI_ISL_2309567
PRJNA686984	SAMN19412145	SRR17435730	OM248995	EPI_ISL_2309568
PRJNA686984	SAMN19412147	SRR17435732	OM249528	EPI_ISL_2309570
PRJNA686984	SAMN19412236	SRR17435496	OM249532	EPI_ISL_2309659

BioProject	BioSample	SRA	GenBank	GISAID
PRJNA686984	SAMN19412239	SRR17435709	OM248685	EPI_ISL_2309662
PRJNA686984	SAMN19412240	SRR17435710	OM249037	EPI_ISL_2309663
PRJNA686984	SAMN19412241	SRR17435492	OM249408	EPI_ISL_2309664
PRJNA686984	SAMN19412329	SRR17435429	Not submitted	EPI_ISL_2309752
PRJNA686984	SAMN19412330	SRR17435438	OM248691	EPI_ISL_2309753
PRJNA686984	SAMN19412422	SRR17436176	OM170433	EPI_ISL_2309845
PRJNA686984	SAMN19412613	SRR17488986	OM211672	EPI_ISL_2311034
PRJNA686984	SAMN19412614	SRR17488987	OM211440	EPI_ISL_2311035
PRJNA686984	SAMN19412619	SRR17488993	OM211958	EPI_ISL_2311040
PRJNA686984	SAMN20925174	SRR15601409	MZ936867	EPI_ISL_3583851
PRJNA686984	SAMN20925185	SRR15601396	MZ936868	EPI_ISL_3583896
PRJNA686984	SAMN20925176	SRR15601407	MZ936869	EPI_ISL_3583894
PRJNA686984	SAMN20925193	SRR15601387	MZ936871	EPI_ISL_3583901
PRJNA686984	SAMN20925192	SRR15601388	MZ936872	EPI_ISL_3583900
PRJNA686984	SAMN20925191	SRR15601390	MZ936873	EPI_ISL_3583899
PRJNA686984	SAMN20925190	SRR15601391	MZ936874	EPI_ISL_3583898
PRJNA686984	SAMN21162030	SRR15693184	OK021986	EPI_ISL_3842472
PRJNA686984	SAMN21162239	SRR15693478	OK022132	EPI_ISL_3842754
PRJNA686984	SAMN21162236	SRR15693482	OK022135	EPI_ISL_3842764
PRJNA686984	SAMN21027455	SRR15699039	OK019930	EPI_ISL_3760581
PRJNA686984	SAMN21209179	SRR15706544	OK075210	EPI_ISL_3916480
PRJNA686984	SAMN21209181	SRR15706542	OK075212	EPI_ISL_3916482
PRJNA686984	SAMN21209182	SRR15706541	OK075213	EPI_ISL_3916483
PRJNA686984	SAMN21209183	SRR15706539	OK075214	EPI_ISL_3916484
PRJNA686984	SAMN21209184	SRR15706538	OK075215	EPI_ISL_3916485
PRJNA686984	SAMN21209185	SRR15706537	OK075216	EPI_ISL_3916486
PRJNA686984	SAMN21209186	SRR15706536	OK075217	EPI_ISL_3916487
PRJNA686984	SAMN21209187	SRR15706535	OK075218	EPI_ISL_3916488
PRJNA686984	SAMN21243525	SRR15899846	OK160293	EPI_ISL_3988606
PRJNA686984	SAMN21243580	SRR15899636	OK160111	EPI_ISL_3988525
PRJNA686984	SAMN21243390	SRR15900191	OK160116	EPI_ISL_3988564
PRJNA686984	SAMN21243522	SRR15899849	OK160423	EPI_ISL_4197811
PRJNA686984	SAMN21243563	SRR15899687	OK160652	EPI_ISL_3988631
PRJNA686984	SAMN21243715	SRR15899959	OK160577	EPI_ISL_3988935
PRJNA686984	SAMN21243788	SRR15899811	OK160183	EPI_ISL_3988565
PRJNA686984	SAMN21243716	SRR15899958	OK160553	EPI_ISL_3988647
PRJNA686984	SAMN21243717	SRR15899957	OK160194	EPI_ISL_3988950
PRJNA686984	SAMN21243718	SRR15899956	OK160135	EPI_ISL_3988547
PRJNA686984	SAMN21243719	SRR15899955	OK160099	EPI_ISL_3988845
PRJNA686984	SAMN21243720	SRR15899953	OK160462	EPI_ISL_3988593
PRJNA686984	SAMN21243723	SRR15899950	OK160520	EPI_ISL_3988537
PRJNA686984	SAMN21243790	SRR15899807	OK160660	EPI_ISL_3988533
PRJNA686984	SAMN21243737	SRR15899935	OK160396	EPI_ISL_3988669
PRJNA686984	SAMN21243714	SRR15899960	OK160620	EPI_ISL_3988862
PRJNA686984	SAMN21243722	SRR15899951	OK160624	EPI_ISL_3988595
PRJNA686984	SAMN21243736	SRR15899936	OK160083	EPI_ISL_3988578
PRJNA686984	SAMN21243725	SRR15899948	OK160339	EPI_ISL_3988851
PRJNA686984	SAMN21243726	SRR15899947	OK160117	EPI_ISL_3988546
PRJNA686984	SAMN21243727	SRR15899946	OK160328	EPI_ISL_3988562
PRJNA686984	SAMN21243728	SRR15899945	OK160522	EPI_ISL_3988685
PRJNA686984	SAMN21243729	SRR15899944	OK160352	EPI_ISL_3988585
PRJNA686984	SAMN21243730	SRR15899942	OK160223	EPI_ISL_3988588
PRJNA686984	SAMN21243724	SRR15899949	OK160471	EPI_ISL_3988677
PRJNA686984	SAMN21243787	SRR15899812	OK160425	EPI_ISL_3988598
PRJNA686984	SAMN21243789	SRR15899810	OK160279	EPI_ISL_3988717
PRJNA686984	SAMN21243733	SRR15899939	OK160221	EPI_ISL_3988872
PRJNA686984	SAMN21243734	SRR15899938	OK160226	EPI_ISL_3988695
PRJNA686984	SAMN21243748	SRR15899923	OK160617	EPI_ISL_3988841
PRJNA686984	SAMN21243735	SRR15899937	OK160101	EPI_ISL_3988665
PRJNA686984	SAMN21243732	SRR15899940	OK160550	EPI_ISL_3988678
PRJNA686984	SAMN21243749	SRR15899922	OK160285	EPI_ISL_3988789
PRJNA686984	SAMN21243721	SRR15899952	OK160458	EPI_ISL_3988865
PRJNA686984	SAMN21380824	SRR15840795	OK267027	EPI_ISL_4207120
PRJNA686984	SAMN21380813	SRR15840808	OK267016	EPI_ISL_4207109
PRJNA686984	SAMN21380866	SRR15840832	OK267067	EPI_ISL_4207160
PRJNA686984	SAMN21380803	SRR15840819	OK267006	EPI_ISL_4207099
PRJNA686984	SAMN21380848	SRR15840852	OK267051	EPI_ISL_4207144
PRJNA686984	SAMN21380853	SRR15840847	OK267055	EPI_ISL_4207148
PRJNA686984	SAMN21380807	SRR15840814	OK267010	EPI_ISL_4207103

BioProject	BioSample	SRA	GenBank	GISAID
PRJNA686984	SAMN21380847	SRR15840853	OK267050	EPI_ISL_4207143
PRJNA686984	SAMN21380851	SRR15840849	OK267053	EPI_ISL_4207146
PRJNA686984	SAMN21380819	SRR15840801	OK267022	EPI_ISL_4207115
PRJNA686984	SAMN21380845	SRR15840855	OK267048	EPI_ISL_4207141
PRJNA686984	SAMN21380802	SRR15840820	OK267005	EPI_ISL_4207098
PRJNA686984	SAMN21380852	SRR15840848	OK267054	EPI_ISL_4207147
PRJNA686984	SAMN21380859	SRR15840840	OK267061	EPI_ISL_4207154
PRJNA686984	SAMN21380809	SRR15840812	OK267012	EPI_ISL_4207105
PRJNA686984	SAMN21380826	SRR15840876	OK267029	EPI_ISL_4207122
PRJNA686984	SAMN21380829	SRR15840873	OK267032	EPI_ISL_4207125
PRJNA686984	SAMN21380791	SRR15840846	OK266994	EPI_ISL_4207088
PRJNA686984	SAMN21380834	SRR15840867	OK267037	EPI_ISL_4207130
PRJNA686984	SAMN21380789	SRR15840868	OK266992	EPI_ISL_4207086
PRJNA686984	SAMN21380857	SRR15840842	OK267059	EPI_ISL_4207152
PRJNA686984	SAMN21380860	SRR15840839	OK267062	EPI_ISL_4207155
PRJNA686984	SAMN21380820	SRR15840800	OK267023	EPI_ISL_4207116
PRJNA686984	SAMN21380867	SRR15840831	OK267068	EPI_ISL_4207161
PRJNA686984	SAMN21380817	SRR15840803	OK267020	EPI_ISL_4207113
PRJNA686984	SAMN21380818	SRR15840802	OK267021	EPI_ISL_4207114
PRJNA686984	SAMN21380858	SRR15840841	OK267060	EPI_ISL_4207153
PRJNA686984	SAMN21380823	SRR15840797	OK267026	EPI_ISL_4207119
PRJNA686984	SAMN21380831	SRR15840871	OK267034	EPI_ISL_4207127
PRJNA686984	SAMN21381172	SRR15843123	OK139657	EPI_ISL_4079406
PRJNA686984	SAMN22120096	SRR16302005	OK483250	EPI_ISL_4963895
PRJNA686984	SAMN22120085	SRR16302017	OK483297	EPI_ISL_4963865
PRJNA686984	SAMN22120098	SRR16302003	OK483274	EPI_ISL_4963899
PRJNA686984	SAMN22120099	SRR16302002	OK483276	EPI_ISL_4963902
PRJNA686984	SAMN22120100	SRR16302001	OK483307	EPI_ISL_4963906
PRJNA686984	SAMN22120158	SRR16301937	OK483275	EPI_ISL_4964074
PRJNA686984	SAMN22120138	SRR16301959	OK483231	EPI_ISL_4964005
PRJNA686984	SAMN22120075	SRR16301999	OK483294	EPI_ISL_4963836
PRJNA686984	SAMN22120074	SRR16302010	OK483288	EPI_ISL_4963833
PRJNA686984	SAMN22120135	SRR16301962	OK483259	EPI_ISL_4963995
PRJNA686984	SAMN22120152	SRR16301943	OK483289	EPI_ISL_4964047
PRJNA686984	SAMN22120115	SRR16301984	OK483295	EPI_ISL_4963941
PRJNA686984	SAMN22120155	SRR16301940	OK483266	EPI_ISL_4964062
PRJNA686984	SAMN22120092	SRR16302009	OK483251	EPI_ISL_4963886
PRJNA686984	SAMN22120126	SRR16301972	OK483235	EPI_ISL_4963968
PRJNA686984	SAMN22120117	SRR16301982	OK483277	EPI_ISL_4963947
PRJNA686984	SAMN22120107	SRR16301993	OK483265	EPI_ISL_4963920
PRJNA686984	SAMN22120095	SRR16302006	OK483238	EPI_ISL_4963894
PRJNA686984	SAMN21889148	SRR16106318	OK349781	EPI_ISL_4574608
PRJNA686984	SAMN21889221	SRR16106556	OK349821	EPI_ISL_4574660
PRJNA686984	SAMN21889173	SRR16106267	OK349824	EPI_ISL_4574698
PRJNA686984	SAMN21889195	SRR16106589	OK349959	EPI_ISL_4574604
PRJNA686984	SAMN21889166	SRR16106275	OK349907	EPI_ISL_4574872
PRJNA686984	SAMN21889176	SRR16106264	OK349830	EPI_ISL_4574702
PRJNA686984	SAMN21889225	SRR16106552	OK350008	EPI_ISL_4574669
PRJNA686984	SAMN21889167	SRR16106274	OK349833	EPI_ISL_4574819
PRJNA686984	SAMN21889209	SRR16106570	OK349780	EPI_ISL_4574631
PRJNA686984	SAMN21889175	SRR16106265	OK349961	EPI_ISL_4574696
PRJNA686984	SAMN21889169	SRR16106272	OK349745	EPI_ISL_4574686
PRJNA686984	SAMN21889205	SRR16106578	OK349892	EPI_ISL_4574710
PRJNA686984	SAMN21889204	SRR16106579	OK349898	EPI_ISL_4574735
PRJNA686984	SAMN21889201	SRR16106582	OK349764	EPI_ISL_4574583
PRJNA686984	SAMN21889140	SRR16106327	OK349857	EPI_ISL_4574657
PRJNA686984	SAMN21889142	SRR16106324	OK349812	EPI_ISL_4574659
PRJNA686984	SAMN21889149	SRR16106317	OK349860	EPI_ISL_4574815
PRJNA686984	SAMN21889180	SRR16106606	OK349910	EPI_ISL_4574688
PRJNA686984	SAMN21889243	SRR16106532	OK349890	EPI_ISL_4574809
PRJNA686984	SAMN21889257	SRR16106517	OK349994	EPI_ISL_4574889
PRJNA686984	SAMN21889280	SRR16106491	OK350047	EPI_ISL_4574723
PRJNA686984	SAMN21889301	SRR16106435	OK349711	EPI_ISL_4634560
PRJNA686984	SAMN21889242	SRR16106533	OK349815	EPI_ISL_4574813
PRJNA686984	SAMN21889282	SRR16106488	OK349856	EPI_ISL_4574775
PRJNA686984	SAMN21889293	SRR16106444	OK349868	EPI_ISL_4574597
PRJNA686984	SAMN21989443	SRR16168179	Not submitted	EPI_ISL_4745522
PRJNA686984	SAMN21989470	SRR16168513	OK385451	EPI_ISL_4745337
PRJNA686984	SAMN21989174	SRR16167902	OK385352	EPI_ISL_4745523

BioProject	BioSample	SRA	GenBank	GISAID
PRJNA686984	SAMN23070493	SRR16918170	OL453636	EPI_ISL_5987668
PRJNA686984	SAMN21989176	SRR16167880	OK385054	EPI_ISL_4745300
PRJNA686984	SAMN23070494	SRR16918169	OL453746	EPI_ISL_5987670
PRJNA686984	SAMN21989179	SRR16167847	OK384943	EPI_ISL_4745118
PRJNA686984	SAMN21989181	SRR16168497	OK385076	EPI_ISL_4745128
PRJNA686984	SAMN21989183	SRR16168443	OK385468	EPI_ISL_4745058
PRJNA686984	SAMN23070496	SRR16918167	OL453355	EPI_ISL_5987672
PRJNA686984	SAMN23414384	SRR17099324	OL702656	EPI_ISL_6678039
PRJNA686984	SAMN21989184	SRR16168431	OK384924	EPI_ISL_4745086
PRJNA686984	SAMN21989187	SRR16168428	OK385285	EPI_ISL_4745023
PRJNA686984	SAMN23070497	SRR16918166	OL453281	EPI_ISL_5987673
PRJNA686984	SAMN21989191	SRR16168424	OK384900	EPI_ISL_4745456
PRJNA686984	SAMN21989194	SRR16168388	OK385410	EPI_ISL_4744934
PRJNA686984	SAMN21989195	SRR16168387	OK385182	EPI_ISL_4745524
PRJNA686984	SAMN21989197	SRR16168385	OK385417	EPI_ISL_4744870
PRJNA686984	SAMN21989172	SRR16167925	OK385419	EPI_ISL_4744854
PRJNA686984	SAMN21989198	SRR16168384	OK385518	EPI_ISL_4744874
PRJNA686984	SAMN21989196	SRR16168386	OK385067	EPI_ISL_4745220
PRJNA686984	SAMN21989192	SRR16168391	OK385280	EPI_ISL_4745252
PRJNA686984	SAMN21989180	SRR16167836	OK385232	EPI_ISL_4744942
PRJNA686984	SAMN21989149	SRR16168520	OK385173	EPI_ISL_4745438
PRJNA686984	SAMN21989273	SRR16168392	OK385362	EPI_ISL_4744975
PRJNA686984	SAMN21989289	SRR16168350	OK385409	EPI_ISL_4744921
PRJNA686984	SAMN21989238	SRR16168462	OK385450	EPI_ISL_4745213
PRJNA686984	SAMN21989281	SRR16168359	OK385053	EPI_ISL_4745457
PRJNA686984	SAMN23070498	SRR16918165	OL453315	EPI_ISL_5987674
PRJNA686984	SAMN23070499	SRR16918164	OL453292	EPI_ISL_5987675
PRJNA686984	SAMN23414549	SRR17099747	OL702308	EPI_ISL_6678209
PRJNA686984	SAMN23070500	SRR16918163	OL453638	EPI_ISL_5987676
PRJNA686984	SAMN23070501	SRR16918162	OL453417	EPI_ISL_5987677
PRJNA686984	SAMN23070502	SRR16918160	OL453967	EPI_ISL_5987678
PRJNA686984	SAMN23070503	SRR16918159	OL453284	EPI_ISL_5987679
PRJNA686984	SAMN23070504	SRR16918158	OL453856	EPI_ISL_5987680
PRJNA686984	SAMN23070505	SRR16918157	OL453622	EPI_ISL_5987681
PRJNA686984	SAMN23070507	SRR16918155	OL453604	EPI_ISL_5987683
PRJNA686984	SAMN21989305	SRR16168332	OK385063	EPI_ISL_4744916
PRJNA686984	SAMN21989302	SRR16168336	OK385277	EPI_ISL_4744968
PRJNA686984	SAMN21989308	SRR16168329	OK385210	EPI_ISL_4744995
PRJNA686984	SAMN23414227	SRR17099571	OL702470	EPI_ISL_6677869
PRJNA686984	SAMN23414336	SRR17099558	OL702322	EPI_ISL_6677988
PRJNA686984	SAMN23414364	SRR17099346	OL702089	EPI_ISL_6678019
PRJNA686984	SAMN23414456	SRR17099156	OL702346	EPI_ISL_6678113
PRJNA686984	SAMN23414329	SRR17099565	OL702448	EPI_ISL_6677981
PRJNA686984	SAMN23414502	SRR17099476	OL702072	EPI_ISL_6678159
PRJNA686984	SAMN23414464	SRR17099147	OL702379	EPI_ISL_6678121
PRJNA686984	SAMN23414397	SRR17099521	OL702191	EPI_ISL_6678052
PRJNA686984	SAMN23414619	SRR17099454	OL702185	EPI_ISL_6678281
PRJNA686984	SAMN23414424	SRR17099316	OL702488	EPI_ISL_6678079
PRJNA686984	SAMN23414358	SRR17099532	OL702353	EPI_ISL_6678010
PRJNA686984	SAMN23414379	SRR17099329	OL702594	EPI_ISL_6678034
PRJNA686984	SAMN23374542	SRR17011805	OL624101	EPI_ISL_6600609
PRJNA686984	SAMN23414271	SRR17099377	OL702392	EPI_ISL_6677915
PRJNA686984	SAMN23414618	SRR17099455	OL702516	EPI_ISL_6678280
PRJNA686984	SAMN23414482	SRR17099858	OL702247	EPI_ISL_6678139
PRJNA686984	SAMN23414455	SRR17099157	OL702472	EPI_ISL_6678112
PRJNA686984	SAMN23414284	SRR17099363	OL702007	EPI_ISL_6677928
PRJNA686984	SAMN23414457	SRR17099155	OL702082	EPI_ISL_6678114
PRJNA686984	SAMN23414386	SRR17099322	OL702607	EPI_ISL_6678041
PRJNA686984	SAMN23414429	SRR17099310	OL702249	EPI_ISL_6678086
PRJNA686984	SAMN23414351	SRR17099540	OL702009	EPI_ISL_6678003
PRJNA686984	SAMN23414660	SRR17099732	OL702655	EPI_ISL_6678324
PRJNA686984	SAMN23414352	SRR17099539	OL702537	EPI_ISL_6678004
PRJNA686984	SAMN23414578	SRR17099252	OL702361	EPI_ISL_6678238
PRJNA686984	SAMN23414267	SRR17099382	OL702068	EPI_ISL_6677911
PRJNA686984	SAMN23414425	SRR17099315	OL702421	EPI_ISL_6678080
PRJNA686984	SAMN23414632	SRR17099440	OL702559	EPI_ISL_6678294
PRJNA686984	SAMN23414493	SRR17099486	OL702338	EPI_ISL_6678150
PRJNA686984	SAMN23414285	SRR17099362	OL702211	EPI_ISL_6677929
PRJNA686984	SAMN23414356	SRR17099535	OL702494	EPI_ISL_6678008

BioProject	BioSample	SRA	GenBank	GISAID
PRJNA686984	SAMN23414298	SRR17099192	OL702039	EPI_ISL_6677942
PRJNA686984	SAMN23414331	SRR17099563	OL702058	EPI_ISL_6677983
PRJNA686984	SAMN23414400	SRR17099517	OL702679	EPI_ISL_6678055
PRJNA686984	SAMN23414350	SRR17099541	OL702672	EPI_ISL_6678002
PRJNA686984	SAMN23414278	SRR17099369	OL702186	EPI_ISL_6677922
PRJNA686984	SAMN23414639	SRR17099431	OL702237	EPI_ISL_6678303
PRJNA686984	SAMN23414316	SRR17099173	OL702561	EPI_ISL_6677968
PRJNA686984	SAMN23373867	SRR17011693	OL624142	EPI_ISL_6599919
PRJNA686984	SAMN23374025	SRR17011707	OL624053	EPI_ISL_6600080
PRJNA686984	SAMN23373927	SRR17012153	OL624514	EPI_ISL_6599979
PRJNA686984	SAMN23373905	SRR17011943	OL623985	EPI_ISL_6599957
PRJNA686984	SAMN23374049	SRR17011679	OL623960	EPI_ISL_6600104
PRJNA686984	SAMN23374219	SRR17011491	OL624014	EPI_ISL_6600278
PRJNA686984	SAMN23374014	SRR17011719	OL624168	EPI_ISL_6600069
PRJNA686984	SAMN23373863	SRR17011737	OL623971	EPI_ISL_6599915
PRJNA686984	SAMN23373990	SRR17011745	OL624000	EPI_ISL_6600045
PRJNA686984	SAMN23373939	SRR17012139	OL624355	EPI_ISL_6599991
PRJNA686984	SAMN23374092	SRR17011632	OL624112	EPI_ISL_6600149
PRJNA686984	SAMN23374213	SRR17011498	OL624340	EPI_ISL_6600272
PRJNA686984	SAMN23374081	SRR17011644	OL624244	EPI_ISL_6600138
PRJNA686984	SAMN23374266	SRR17012111	OL624013	EPI_ISL_6600325
PRJNA686984	SAMN23374164	SRR17011552	OL624427	EPI_ISL_6600221
PRJNA686984	SAMN23374371	SRR17011994	OL623898	EPI_ISL_6600432
PRJNA686984	SAMN23373873	SRR17011626	OL624375	EPI_ISL_6599925
PRJNA686984	SAMN23373870	SRR17011659	OL624304	EPI_ISL_6599922
PRJNA686984	SAMN23374062	SRR17011665	OL624313	EPI_ISL_6600117
PRJNA686984	SAMN23374056	SRR17011672	OL624403	EPI_ISL_6600111
PRJNA686984	SAMN23374482	SRR17011871	OL624323	EPI_ISL_6600546
PRJNA686984	SAMN23373897	SRR17012032	OL623974	EPI_ISL_6599949
PRJNA686984	SAMN23373852	SRR17011460	OL624018	EPI_ISL_6599904
PRJNA686984	SAMN22251486	SRR16322844	OK596737	EPI_ISL_5154950
PRJNA686984	SAMN23373859	SRR17011781	OL624072	EPI_ISL_6599911
PRJNA686984	SAMN23374280	SRR17012095	OL623938	EPI_ISL_6600339
PRJNA686984	SAMN23374441	SRR17011917	OL624132	EPI_ISL_6600505
PRJNA686984	SAMN23373861	SRR17011759	OL624146	EPI_ISL_6599913
PRJNA686984	SAMN23374018	SRR17011714	OL624384	EPI_ISL_6600073
PRJNA686984	SAMN23374443	SRR17011915	OL624208	EPI_ISL_6600507
PRJNA686984	SAMN23373868	SRR17011681	OL624087	EPI_ISL_6599920
PRJNA686984	SAMN23374142	SRR17011577	OL623871	EPI_ISL_6600199
PRJNA686984	SAMN23374362	SRR17012004	OL624422	EPI_ISL_6600423
PRJNA686984	SAMN23373909	SRR17011898	OL623941	EPI_ISL_6599961
PRJNA686984	SAMN23374356	SRR17012011	Not submitted	EPI_ISL_6600417
PRJNA686984	SAMN23374078	SRR17011647	OL624189	EPI_ISL_6600135
PRJNA686984	SAMN23373862	SRR17011748	OL624285	EPI_ISL_6599914
PRJNA686984	SAMN23374528	SRR17011820	OL624505	EPI_ISL_6600595
PRJNA686984	SAMN23374104	SRR17011619	OL624292	EPI_ISL_6600161
PRJNA686984	SAMN23373935	SRR17012144	OL624246	EPI_ISL_6599987
PRJNA686984	SAMN23373884	SRR17011504	OL624184	EPI_ISL_6599936
PRJNA686984	SAMN23373906	SRR17011932	OL624275	EPI_ISL_6599958
PRJNA686984	SAMN23373855	SRR17011799	OL624120	EPI_ISL_6599907
PRJNA686984	SAMN23374531	SRR17011817	OL624245	EPI_ISL_6600598
PRJNA686984	SAMN23373856	SRR17012152	OL624309	EPI_ISL_6599908
PRJNA686984	SAMN21622391	SRR16070969	OK298204	EPI_ISL_4507230
PRJNA686984	SAMN21622392	SRR16070968	OK298169	EPI_ISL_4507231
PRJNA686984	SAMN21622647	SRR16071044	OK298343	EPI_ISL_4507485
PRJNA686984	SAMN21622456	SRR16070896	OK298075	EPI_ISL_4507295
PRJNA686984	SAMN21622457	SRR16070895	OK298238	EPI_ISL_4507296
PRJNA686984	SAMN21622459	SRR16070893	OK298338	EPI_ISL_4507298
PRJNA686984	SAMN21622323	SRR16071117	OK297734	EPI_ISL_4507162
PRJNA686984	SAMN21622460	SRR16070892	OK298041	EPI_ISL_4507299
PRJNA686984	SAMN21622462	SRR16070890	OK297918	EPI_ISL_4507301
PRJNA686984	SAMN21622464	SRR16070888	OK298293	EPI_ISL_4507303
PRJNA686984	SAMN21622468	SRR16070883	OK298048	EPI_ISL_4507307
PRJNA686984	SAMN21622469	SRR16070882	OK297857	EPI_ISL_4507308
PRJNA686984	SAMN21622470	SRR16070881	OK298072	EPI_ISL_4507309
PRJNA686984	SAMN21622331	SRR16071076	OK297721	EPI_ISL_4507170
PRJNA686984	SAMN21622484	SRR16070866	OK298159	EPI_ISL_4507323
PRJNA686984	SAMN21622483	SRR16070867	OK297830	EPI_ISL_4507322
PRJNA686984	SAMN21622482	SRR16070868	OK298324	EPI_ISL_4507321

BioProject	BioSample	SRA	GenBank	GISAID
PRJNA686984	SAMN21622487	SRR16070862	OK298374	EPI_ISL_4507326
PRJNA686984	SAMN21622335	SRR16071072	OK297840	EPI_ISL_4507174
PRJNA686984	SAMN21622478	SRR16070872	OK298316	EPI_ISL_4507317
PRJNA686984	SAMN21622310	SRR16071131	OK298303	EPI_ISL_4507149
PRJNA686984	SAMN21622477	SRR16070873	OK297805	EPI_ISL_4507316
PRJNA686984	SAMN21622304	SRR16070466	OK298163	EPI_ISL_4507143
PRJNA686984	SAMN21622476	SRR16070874	OK297688	EPI_ISL_4507315
PRJNA686984	SAMN21622473	SRR16070878	OK297733	EPI_ISL_4507312
PRJNA686984	SAMN21622366	SRR16071004	OK297710	EPI_ISL_4507205
PRJNA686984	SAMN21622307	SRR16070462	OK298138	EPI_ISL_4507146
PRJNA686984	SAMN21622308	SRR16071133	OK298037	EPI_ISL_4507147
PRJNA686984	SAMN22581793	SRR16645304	OL317694	EPI_ISL_5607685
PRJNA686984	SAMN22581794	SRR16645303	OL318336	EPI_ISL_5607599
PRJNA686984	SAMN22581795	SRR16645302	OL318276	EPI_ISL_5607155
PRJNA686984	SAMN22581796	SRR16645301	OL318095	EPI_ISL_5605305
PRJNA686984	SAMN22581797	SRR16645299	OL318196	EPI_ISL_5609753
PRJNA686984	SAMN22581798	SRR16645298	OL317978	EPI_ISL_5607525
PRJNA686984	SAMN22581799	SRR16645322	OL318208	EPI_ISL_5608856
PRJNA686984	SAMN22581804	SRR16645292	OL318269	EPI_ISL_5608236
PRJNA686984	SAMN22581809	SRR16645311	OL317946	EPI_ISL_5605566
PRJNA686984	SAMN22581812	SRR16645261	OL318245	EPI_ISL_5607373
PRJNA686984	SAMN22581813	SRR16645259	OL317984	EPI_ISL_5604935
PRJNA686984	SAMN22581815	SRR16645282	OL318403	EPI_ISL_5606413
PRJNA686984	SAMN22581824	SRR16645272	OL317935	EPI_ISL_5604939
PRJNA686984	SAMN22581825	SRR16645271	OL317815	EPI_ISL_5607714
PRJNA686984	SAMN22581829	SRR16645265	OL317989	EPI_ISL_5607072
PRJNA686984	SAMN22581830	SRR16645263	OL318101	EPI_ISL_5609577
PRJNA686984	SAMN22581832	SRR16645232	OL318106	EPI_ISL_5608577
PRJNA686984	SAMN22581833	SRR16645231	OL318324	EPI_ISL_5606374
PRJNA686984	SAMN22581834	SRR16645258	OL317855	EPI_ISL_5606095
PRJNA686984	SAMN22581835	SRR16645257	OL318221	EPI_ISL_5607025
PRJNA686984	SAMN22581836	SRR16645256	OL318171	EPI_ISL_5608906
PRJNA686984	SAMN22581837	SRR16645254	OL317769	EPI_ISL_5608784
PRJNA686984	SAMN22581838	SRR16645253	OL317736	EPI_ISL_5608993
PRJNA686984	SAMN22581839	SRR16645252	OL318110	EPI_ISL_5606107
PRJNA686984	SAMN22581840	SRR16645250	OL318305	EPI_ISL_5607940
PRJNA686984	SAMN22581841	SRR16645248	OL318375	EPI_ISL_5606328
PRJNA686984	SAMN22581842	SRR16645247	OL317830	EPI_ISL_5606842
PRJNA686984	SAMN22581843	SRR16645246	Not submitted	EPI_ISL_5605106
PRJNA686984	SAMN22581844	SRR16645243	OL317763	EPI_ISL_5607114
PRJNA686984	SAMN22581845	SRR16645242	OL317957	EPI_ISL_5607141
PRJNA686984	SAMN22581846	SRR16645241	OL318160	EPI_ISL_5608414
PRJNA686984	SAMN22581847	SRR16645240	OL318115	EPI_ISL_5607250
PRJNA686984	SAMN22581848	SRR16645267	OL317664	EPI_ISL_5605688
PRJNA686984	SAMN22581849	SRR16645266	OL317856	EPI_ISL_5608871
PRJNA686984	SAMN22581850	SRR16645238	OL317726	EPI_ISL_5607878
PRJNA686984	SAMN22581851	SRR16645237	OL318138	EPI_ISL_5606320
PRJNA686984	SAMN22581852	SRR16645236	OL317988	EPI_ISL_5608103
PRJNA686984	SAMN22581853	SRR16645234	OL318043	EPI_ISL_5607099
PRJNA686984	SAMN22581959	SRR16645586	OL317859	EPI_ISL_5608542
PRJNA686984	SAMN22581960	SRR16645558	OL318367	EPI_ISL_5605551
PRJNA686984	SAMN22581961	SRR16645557	OL318345	EPI_ISL_5605929
PRJNA686984	SAMN22581962	SRR16645556	OL317746	EPI_ISL_5606983
PRJNA686984	SAMN22581963	SRR16645555	OL317768	EPI_ISL_5606451
PRJNA686984	SAMN22581964	SRR16645554	OL317791	EPI_ISL_5606240
PRJNA686984	SAMN22581965	SRR16645552	OL318219	EPI_ISL_5608795
PRJNA686984	SAMN22581966	SRR16645551	OL317758	EPI_ISL_5608931
PRJNA686984	SAMN22581968	SRR16645576	OL317651	EPI_ISL_5608701
PRJNA686984	SAMN22581969	SRR16645575	OL317896	EPI_ISL_5609086
PRJNA686984	SAMN22581970	SRR16645548	OL317676	EPI_ISL_5606744
PRJNA686984	SAMN22581971	SRR16645520	OL318314	EPI_ISL_5609754
PRJNA686984	SAMN22581972	SRR16645519	OL317952	EPI_ISL_5606080
PRJNA686984	SAMN22581973	SRR16645518	OL318156	EPI_ISL_5608636
PRJNA686984	SAMN22581974	SRR16645517	OL317705	EPI_ISL_5607851
PRJNA686984	SAMN22581975	SRR16645545	OL317819	EPI_ISL_5605164
PRJNA686984	SAMN22581976	SRR16645544	OL317818	EPI_ISL_5605576
PRJNA686984	SAMN22581977	SRR16645541	OL317790	EPI_ISL_5605963
PRJNA686984	SAMN22581978	SRR16645540	OL318197	EPI_ISL_5607188
PRJNA686984	SAMN22581979	SRR16645564	OL317977	EPI_ISL_5609716

BioProject	BioSample	SRA	GenBank	GISAID
PRJNA686984	SAMN22581981	SRR16645537	OL317779	EPI_ISL_5607127
PRJNA686984	SAMN22581982	SRR16645535	OL317757	EPI_ISL_5609764
PRJNA686984	SAMN22581983	SRR16645534	OL317719	EPI_ISL_5606251
PRJNA686984	SAMN22581984	SRR16645533	OL317788	EPI_ISL_5606265
PRJNA686984	SAMN22581985	SRR16645530	OL317974	EPI_ISL_5608935
PRJNA686984	SAMN22581987	SRR16645528	OL318388	EPI_ISL_5607582
PRJNA686984	SAMN22581988	SRR16645527	OL317686	EPI_ISL_5607880
PRJNA686984	SAMN22581989	SRR16645553	OL318097	EPI_ISL_5607888
PRJNA686984	SAMN22581992	SRR16645522	OL318340	EPI_ISL_5607136
PRJNA686984	SAMN22581993	SRR16645521	OL317981	EPI_ISL_5606791
PRJNA686984	SAMN22581994	SRR16645547	OL317811	EPI_ISL_5607668
PRJNA686984	SAMN22581996	SRR16645157	OL317656	EPI_ISL_5604877
PRJNA686984	SAMN22581997	SRR16645516	OL318119	EPI_ISL_5607086
PRJNA686984	SAMN22581999	SRR16645542	OL317744	EPI_ISL_5609037
PRJNA686984	SAMN22582000	SRR16645513	OL318027	EPI_ISL_5604905
PRJNA686984	SAMN22582001	SRR16645512	OL318175	EPI_ISL_5607381
PRJNA686984	SAMN22582002	SRR16645175	OL317728	EPI_ISL_5605948
PRJNA686984	SAMN22582004	SRR16645536	OL318072	EPI_ISL_5604943
PRJNA686984	SAMN22582006	SRR16645172	OL318033	EPI_ISL_5606396
PRJNA686984	SAMN22582008	SRR16645532	OL318283	EPI_ISL_5606403
PRJNA686984	SAMN22582009	SRR16645531	OL318065	EPI_ISL_5607343
PRJNA686984	SAMN22582011	SRR16645168	OL318155	EPI_ISL_5609506
PRJNA686984	SAMN22582012	SRR16645167	OL318089	EPI_ISL_5605460
PRJNA686984	SAMN22582013	SRR16645166	OL318127	EPI_ISL_5608623
PRJNA686984	SAMN22582014	SRR16645525	OL318228	EPI_ISL_5606421
PRJNA686984	SAMN22582015	SRR16645165	OL317756	EPI_ISL_5604928
PRJNA686984	SAMN22582017	SRR16645163	OL317737	EPI_ISL_5606309
PRJNA686984	SAMN22582019	SRR16645161	OL317816	EPI_ISL_5604949
PRJNA686984	SAMN22582020	SRR16645101	OL317914	EPI_ISL_5608428
PRJNA686984	SAMN22582021	SRR16645100	OL317881	EPI_ISL_5605983
PRJNA686984	SAMN22582023	SRR16645514	OL318181	EPI_ISL_5607286
PRJNA686984	SAMN22582024	SRR16645154	OL318166	EPI_ISL_5604907
PRJNA686984	SAMN22582026	SRR16645152	OL317891	EPI_ISL_5607881
PRJNA686984	SAMN22582028	SRR16645150	OL317922	EPI_ISL_5604979
PRJNA686984	SAMN22582030	SRR16645147	OL317847	EPI_ISL_5605508
PRJNA686984	SAMN22582031	SRR16645146	OL317774	EPI_ISL_5606422
PRJNA686984	SAMN22582037	SRR16645140	OL318146	EPI_ISL_5609436
PRJNA686984	SAMN22582039	SRR16645102	OL317883	EPI_ISL_5604950
PRJNA686984	SAMN22582040	SRR16645077	OL318333	EPI_ISL_5606660
PRJNA686984	SAMN22582042	SRR16645075	OL317915	EPI_ISL_5606423
PRJNA686984	SAMN22582050	SRR16645091	OL318288	EPI_ISL_5608581
PRJNA686984	SAMN22582052	SRR16645089	OL318301	EPI_ISL_5608810
PRJNA686984	SAMN22582055	SRR16645086	OL317738	EPI_ISL_5606216
PRJNA686984	SAMN22582058	SRR16645083	OL318125	EPI_ISL_5609545
PRJNA686984	SAMN22582059	SRR16645082	OL318302	EPI_ISL_5605583
PRJNA686984	SAMN22582060	SRR16645079	OL317917	EPI_ISL_5604921
PRJNA686984	SAMN21903914	SRR16121250	OK397078	EPI_ISL_4635964
PRJNA686984	SAMN21903901	SRR16121265	OK397065	EPI_ISL_4635951
PRJNA686984	SAMN21903875	SRR16121293	OK397039	EPI_ISL_4635925
PRJNA686984	SAMN21903907	SRR16121258	OK397071	EPI_ISL_4635957
PRJNA686984	SAMN21903936	SRR16121226	OK397098	EPI_ISL_4635987
PRJNA686984	SAMN21903882	SRR16121285	OK397046	EPI_ISL_4635932
PRJNA686984	SAMN22252034	SRR16322947	OK596761	EPI_ISL_5155514
PRJNA686984	SAMN21903853	SRR16121308	OK397017	EPI_ISL_4635903
PRJNA686984	SAMN22252035	SRR16322945	OK596704	EPI_ISL_5155515
PRJNA686984	SAMN22252036	SRR16322944	OK596662	EPI_ISL_5155516
PRJNA686984	SAMN22252037	SRR16322943	OK596836	EPI_ISL_5155517
PRJNA686984	SAMN21903892	SRR16121274	OK397056	EPI_ISL_4635942
PRJNA686984	SAMN22252038	SRR16322942	OK596993	EPI_ISL_5155518
PRJNA686984	SAMN22252039	SRR16322941	OK596759	EPI_ISL_5155519
PRJNA686984	SAMN22252040	SRR16322940	OK596605	EPI_ISL_5155520
PRJNA686984	SAMN21903878	SRR16121290	OK397042	EPI_ISL_4635928
PRJNA686984	SAMN22252041	SRR16322939	OK596697	EPI_ISL_5155521
PRJNA686984	SAMN21903891	SRR16121276	OK397055	EPI_ISL_4635941
PRJNA686984	SAMN22252042	SRR16322938	OK596598	EPI_ISL_5155522
PRJNA686984	SAMN21903920	SRR16121244	Not submitted	EPI_ISL_4703433
PRJNA686984	SAMN22252043	SRR16322937	OK596996	EPI_ISL_5155523
PRJNA686984	SAMN22252045	SRR16322934	OK596974	EPI_ISL_5155525
PRJNA686984	SAMN21904273	SRR16122321	Not submitted	EPI_ISL_4644314

BioProject	BioSample	SRA	GenBank	GISAID
PRJNA686984	SAMN22252047	SRR16322932	OK596992	EPI_ISL_5155527
PRJNA686984	SAMN21903925	SRR16121238	OK397088	EPI_ISL_4635974
PRJNA686984	SAMN22252048	SRR16322931	OK596800	EPI_ISL_5155528
PRJNA686984	SAMN21903896	SRR16121270	OK397060	EPI_ISL_4635946
PRJNA686984	SAMN21903928	SRR16121235	OK397091	EPI_ISL_4635977
PRJNA686984	SAMN21903864	SRR16121305	OK397028	EPI_ISL_4635914
PRJNA686984	SAMN21903941	SRR16121221	OK397103	EPI_ISL_4635992
PRJNA686984	SAMN21904222	SRR16122328	OK396768	EPI_ISL_4625207
PRJNA686984	SAMN21904245	SRR16122352	OK396791	EPI_ISL_4625230
PRJNA686984	SAMN21904230	SRR16122368	OK396776	EPI_ISL_4625215
PRJNA686984	SAMN21904263	SRR16122332	OK396809	EPI_ISL_4625248
PRJNA686984	SAMN21904282	SRR16122315	OK396826	EPI_ISL_4625265
PRJNA686984	SAMN21903918	SRR16121246	OK397082	EPI_ISL_4635968
PRJNA686984	SAMN21904243	SRR16122354	OK396789	EPI_ISL_4625228
PRJNA686984	SAMN21904277	SRR16122376	Not submitted	EPI_ISL_4644326
PRJNA686984	SAMN21904251	SRR16122345	OK396797	EPI_ISL_4625236
PRJNA686984	SAMN21904284	SRR16122313	OK396827	EPI_ISL_4625266
PRJNA686984	SAMN21904268	SRR16122326	OK396814	EPI_ISL_4625253
PRJNA686984	SAMN21904241	SRR16122356	OK396787	EPI_ISL_4625226
PRJNA686984	SAMN21904248	SRR16122348	OK396794	EPI_ISL_4625233
PRJNA686984	SAMN21904249	SRR16122347	OK396795	EPI_ISL_4625234
PRJNA686984	SAMN21904281	SRR16122316	OK396825	EPI_ISL_4625264
PRJNA686984	SAMN21904232	SRR16122366	OK396778	EPI_ISL_4625217
PRJNA686984	SAMN21904220	SRR16122350	OK396766	EPI_ISL_4625205
PRJNA686984	SAMN22252050	SRR16322929	OK596603	EPI_ISL_5155530
PRJNA686984	SAMN21904231	SRR16122367	OK396777	EPI_ISL_4625216
PRJNA686984	SAMN21904261	SRR16122334	OK396807	EPI_ISL_4625246
PRJNA686984	SAMN21904278	SRR16122375	OK396822	EPI_ISL_4625261
PRJNA686984	SAMN21904227	SRR16122371	OK396773	EPI_ISL_4625212
PRJNA686984	SAMN21904219	SRR16122361	OK396765	EPI_ISL_4625204
PRJNA686984	SAMN21904270	SRR16122324	OK396816	EPI_ISL_4625255
PRJNA686984	SAMN21903881	SRR16121287	OK397045	EPI_ISL_4635931
PRJNA686984	SAMN21904224	SRR16122312	OK396770	EPI_ISL_4625209
PRJNA686984	SAMN21904236	SRR16122362	OK396782	EPI_ISL_4625221
PRJNA686984	SAMN21904262	SRR16122333	OK396808	EPI_ISL_4625247
PRJNA686984	SAMN21844802	SRR16090194	OK354752	EPI_ISL_4518588
PRJNA686984	SAMN21844784	SRR16089976	OK354934	EPI_ISL_4518702
PRJNA686984	SAMN21844813	SRR16090182	OK355029	EPI_ISL_4518585
PRJNA686984	SAMN21844922	SRR16090253	OK354979	EPI_ISL_4518375
PRJNA686984	SAMN21844800	SRR16090196	OK354772	EPI_ISL_4518172
PRJNA686984	SAMN22355701	SRR16552525	OK639271	EPI_ISL_5291111
PRJNA686984	SAMN22355161	SRR16377834	OK595979	EPI_ISL_5284055
PRJNA686984	SAMN22304193	SRR16328721	OK590214	EPI_ISL_5157805
PRJNA686984	SAMN22416085	SRR16913676	OL718815	EPI_ISL_5347690
PRJNA686984	SAMN22416072	SRR16913722	OL719045	EPI_ISL_5347677
PRJNA686984	SAMN22415896	SRR16913646	OL718858	EPI_ISL_5347496
PRJNA686984	SAMN22415837	SRR16913870	OL718882	EPI_ISL_5347437
PRJNA686984	SAMN22415840	SRR16913710	OL718850	EPI_ISL_5347440
PRJNA686984	SAMN22415847	SRR16913868	OL718887	EPI_ISL_5347447
PRJNA686984	SAMN22868228	SRR16781486	OL407161	EPI_ISL_5931817
PRJNA686984	SAMN22868231	SRR16781483	OL407753	EPI_ISL_5931820
PRJNA686984	SAMN22868237	SRR16781476	OL407807	EPI_ISL_5931826
PRJNA686984	SAMN22868239	SRR16781474	OL407465	EPI_ISL_5931828
PRJNA686984	SAMN22868241	SRR16781472	OL407471	EPI_ISL_5931830
PRJNA686984	SAMN22868242	SRR16781471	OL407463	EPI_ISL_5931831
PRJNA686984	SAMN22868243	SRR16781470	OL407578	EPI_ISL_5931832
PRJNA686984	SAMN22868248	SRR16781463	OL407548	EPI_ISL_5931837
PRJNA686984	SAMN22868249	SRR16781462	OL407483	EPI_ISL_5931838
PRJNA686984	SAMN22868251	SRR16781460	OL407563	EPI_ISL_5931842
PRJNA686984	SAMN22868253	SRR16781458	OL407464	EPI_ISL_5931844
PRJNA686984	SAMN22868254	SRR16781456	OL407262	EPI_ISL_5931845
PRJNA686984	SAMN22868255	SRR16781455	OL407469	EPI_ISL_5931846
PRJNA686984	SAMN22868256	SRR16781454	OL407273	EPI_ISL_5931847
PRJNA686984	SAMN22868257	SRR16781453	OL407977	EPI_ISL_5931848
PRJNA686984	SAMN22868258	SRR16781452	OL407443	EPI_ISL_5931849
PRJNA686984	SAMN23287139	SRR16984597	OL661197	EPI_ISL_6585540
PRJNA686984	SAMN23287166	SRR16984604	OL661223	EPI_ISL_6585567
PRJNA686984	SAMN23287172	SRR16984593	OL661229	EPI_ISL_6585573
PRJNA686984	SAMN23287174	SRR16984591	OL661231	EPI_ISL_6585575

BioProject	BioSample	SRA	GenBank	GISAID
PRJNA686984	SAMN23287152	SRR16984619	OL661209	EPI_ISL_6585553
PRJNA686984	SAMN23287171	SRR16984594	OL661228	EPI_ISL_6585572
PRJNA686984	SAMN23287141	SRR16984631	OL661199	EPI_ISL_6585542
PRJNA686984	SAMN23287188	SRR16984575	OL661245	EPI_ISL_6585589
PRJNA686984	SAMN23287151	SRR16984620	OL661208	EPI_ISL_6585552
PRJNA686984	SAMN23287158	SRR16984612	OL661215	EPI_ISL_6585559
PRJNA686984	SAMN23287196	SRR16984567	OL661253	EPI_ISL_6585597
PRJNA686984	SAMN23287173	SRR16984592	OL661230	EPI_ISL_6585574
PRJNA686984	SAMN23287154	SRR16984617	OL661211	EPI_ISL_6585555
PRJNA686984	SAMN23287201	SRR16984561	OL661258	EPI_ISL_6585602
PRJNA686984	SAMN23287181	SRR16984583	OL661238	EPI_ISL_6585582
PRJNA686984	SAMN23287157	SRR16984614	OL661214	EPI_ISL_6585558
PRJNA686984	SAMN23287164	SRR16984606	OL661221	EPI_ISL_6585565
PRJNA686984	SAMN23287162	SRR16984608	OL661219	EPI_ISL_6585563
PRJNA686984	SAMN23287160	SRR16984610	OL661217	EPI_ISL_6585561
PRJNA686984	SAMN23287165	SRR16984605	OL661222	EPI_ISL_6585566
PRJNA686984	SAMN23287137	SRR16984558	OL661195	EPI_ISL_6585538
PRJNA686984	SAMN23287159	SRR16984611	OL661216	EPI_ISL_6585560
PRJNA686984	SAMN23287140	SRR16984632	OL661198	EPI_ISL_6585541
PRJNA686984	SAMN23287146	SRR16984626	OL661204	EPI_ISL_6585547
PRJNA686984	SAMN23287150	SRR16984621	OL661207	EPI_ISL_6585551
PRJNA686984	SAMN23287136	SRR16984559	OL661194	EPI_ISL_6585537
PRJNA686984	SAMN23287145	SRR16984627	OL661203	EPI_ISL_6585546
PRJNA686984	SAMN23287163	SRR16984607	OL661220	EPI_ISL_6585564
PRJNA686984	SAMN23287182	SRR16984582	OL661239	EPI_ISL_6585583
PRJNA686984	SAMN23287187	SRR16984577	OL661244	EPI_ISL_6585588
PRJNA686984	SAMN23287169	SRR16984596	OL661226	EPI_ISL_6585570
PRJNA686984	SAMN23287197	SRR16984566	OL661254	EPI_ISL_6585598
PRJNA686984	SAMN23287186	SRR16984578	OL661243	EPI_ISL_6585587
PRJNA686984	SAMN23287132	SRR16984602	OL661190	EPI_ISL_6585533
PRJNA686984	SAMN23287130	SRR16984624	OL661188	EPI_ISL_6585531
PRJNA686984	SAMN23287168	SRR16984601	OL661225	EPI_ISL_6585569
PRJNA686984	SAMN23287185	SRR16984579	OL661242	EPI_ISL_6585586
PRJNA686984	SAMN23287155	SRR16984616	OL661212	EPI_ISL_6585556
PRJNA686984	SAMN23287184	SRR16984580	OL661241	EPI_ISL_6585585
PRJNA686984	SAMN23287176	SRR16984589	OL661233	EPI_ISL_6585577
PRJNA686984	SAMN23287180	SRR16984584	OL661237	EPI_ISL_6585581
PRJNA686984	SAMN23287161	SRR16984609	OL661218	EPI_ISL_6585562
PRJNA686984	SAMN23287202	SRR16984560	OL661259	EPI_ISL_6585603
PRJNA686984	SAMN23287142	SRR16984630	OL661200	EPI_ISL_6585543
PRJNA686984	SAMN23287144	SRR16984628	OL661202	EPI_ISL_6585545
PRJNA686984	SAMN23455988	SRR17052257	OL696308	EPI_ISL_6815258
PRJNA686984	SAMN24005312	SRR17268812	OL924602	EPI_ISL_7333948
PRJNA686984	SAMN24005169	SRR17268488	OL924733	EPI_ISL_7333810
PRJNA686984	SAMN24005231	SRR17268743	OL924658	EPI_ISL_7333869
PRJNA686984	SAMN24005330	SRR17268793	OL924732	EPI_ISL_7333966
PRJNA686984	SAMN24005237	SRR17268737	OL925149	EPI_ISL_7333875
PRJNA686984	SAMN24005163	SRR17268494	OL924887	EPI_ISL_7333805
PRJNA686984	SAMN24005194	SRR17269030	OL925022	EPI_ISL_7333835
PRJNA686984	SAMN24005500	SRR17268697	OL924767	EPI_ISL_7334132
PRJNA686984	SAMN24005258	SRR17268570	OL924429	EPI_ISL_7333897
PRJNA686984	SAMN24005174	SRR17268482	OL924957	EPI_ISL_7333815
PRJNA686984	SAMN24004958	SRR17268651	OL924584	EPI_ISL_7333593
PRJNA686984	SAMN24005461	SRR17268523	OL924528	EPI_ISL_7334094
PRJNA686984	SAMN24005200	SRR17269024	OL924570	EPI_ISL_7333841
PRJNA686984	SAMN24005303	SRR17268822	OL924804	EPI_ISL_7333939
PRJNA686984	SAMN24004821	SRR17268989	OL924448	EPI_ISL_7333458
PRJNA686984	SAMN24004943	SRR17268667	OL925130	EPI_ISL_7333579
PRJNA686984	SAMN24005286	SRR17268841	OL924842	EPI_ISL_7333922
PRJNA686984	SAMN24004802	SRR17268486	OL924454	EPI_ISL_7333440
PRJNA686984	SAMN24004816	SRR17268814	OL924955	EPI_ISL_7333454
PRJNA686984	SAMN19408143	SRR17001999	Not Submitted	EPI_ISL_1539306
PRJNA686984	SAMN19408145	SRR17001629	OL891212	EPI_ISL_1540307
PRJNA686984	SAMN19408601	SRR17350568	OM067303	EPI_ISL_1540392
PRJNA686984	SAMN19408653	SRR17350207	OM067553	EPI_ISL_1540399
PRJNA686984	SAMN19404111	SRR17350295	Not submitted	EPI_ISL_2194779
PRJNA686984	SAMN23895374	SRR17201433	OL820384	EPI_ISL_7501688
PRJNA686984	SAMN23895017	SRR17201361	OL820458	EPI_ISL_7501318
PRJNA686984	SAMN23895468	SRR17201401	OL820188	EPI_ISL_7501786

BioProject	BioSample	SRA	GenBank	GISAID
PRJNA686984	SAMN23894806	SRR17201826	OL820031	EPI_ISL_7501096
PRJNA686984	SAMN23894814	SRR17201377	OL820001	EPI_ISL_7501104
PRJNA686984	SAMN23895502	SRR17201197	OL820487	EPI_ISL_7501821
PRJNA686984	SAMN23894885	SRR17201231	OL820497	EPI_ISL_7501179
PRJNA686984	SAMN23895177	SRR17201580	OL820349	EPI_ISL_7501483
PRJNA686984	SAMN23895535	SRR17201160	OL820643	EPI_ISL_7501854
PRJNA686984	SAMN23895527	SRR17201169	OL820395	EPI_ISL_7501846
PRJNA686984	SAMN23895147	SRR17201613	OL820713	EPI_ISL_7501452
PRJNA686984	SAMN23894816	SRR17201355	OL820411	EPI_ISL_7501106
PRJNA686984	SAMN23895439	SRR17201253	OL820466	EPI_ISL_7501755
PRJNA686984	SAMN23895318	SRR17201351	OL819984	EPI_ISL_7501628
PRJNA686984	SAMN23895348	SRR17201318	OL820566	EPI_ISL_7501658
PRJNA686984	SAMN23894956	SRR17201681	OL820634	EPI_ISL_7501254
PRJNA686984	SAMN23894853	SRR17201304	OL820412	EPI_ISL_7501147
PRJNA686984	SAMN23894797	SRR17201563	OL820418	EPI_ISL_7501088
PRJNA686984	SAMN23895524	SRR17201172	OL820637	EPI_ISL_7501843
PRJNA686984	SAMN23894813	SRR17201388	OL820628	EPI_ISL_7501103
PRJNA686984	SAMN23895269	SRR17201478	OL820171	EPI_ISL_7501579
PRJNA686984	SAMN23894896	SRR17201855	OL820294	EPI_ISL_7501195
PRJNA686984	SAMN23894851	SRR17201435	OL820379	EPI_ISL_7501145
PRJNA686984	SAMN23895485	SRR17201216	OL820306	EPI_ISL_7501803
PRJNA686984	SAMN23894976	SRR17201803	OL820055	EPI_ISL_7501277
PRJNA686984	SAMN23895427	SRR17201266	OL820548	EPI_ISL_7501743
PRJNA686984	SAMN23894902	SRR17201849	OL819985	EPI_ISL_7501200
PRJNA686984	SAMN23895411	SRR17201284	OL820327	EPI_ISL_7501727
PRJNA686984	SAMN24003315	SRR17220267	OL842879	EPI_ISL_7332711
PRJNA686984	SAMN24003458	SRR17220322	OL842876	EPI_ISL_7332848
PRJNA686984	SAMN24003743	SRR17219866	OL842741	EPI_ISL_7333126
PRJNA686984	SAMN24003438	SRR17219665	OL842438	EPI_ISL_7332830
PRJNA686984	SAMN24003365	SRR17219709	OL842946	EPI_ISL_7332760
PRJNA686984	SAMN24003561	SRR17219632	Not submitted	Not submitted
PRJNA686984	SAMN24003345	SRR17219451	OL842336	EPI_ISL_7332740
PRJNA686984	SAMN24003434	SRR17219669	OL842315	EPI_ISL_7332827
PRJNA686984	SAMN24003619	SRR17219394	OL843071	EPI_ISL_7333006
PRJNA686984	SAMN24003278	SRR17219784	OL842572	EPI_ISL_7332671
PRJNA686984	SAMN24003336	SRR17219461	OL842479	EPI_ISL_7332731
PRJNA686984	SAMN24003369	SRR17219705	OL842641	EPI_ISL_7332764
PRJNA686984	SAMN24003306	SRR17220278	OL843070	EPI_ISL_7332699
PRJNA686984	SAMN24003970	SRR17220099	OL842286	EPI_ISL_7333346
PRJNA686984	SAMN24003270	SRR17219793	OL842921	EPI_ISL_7332663
PRJNA686984	SAMN24003774	SRR17220177	OL842753	EPI_ISL_7333157
PRJNA686984	SAMN24004012	SRR17220339	OL842845	EPI_ISL_7333387
PRJNA686984	SAMN24003668	SRR17219879	OL842256	EPI_ISL_7333051
PRJNA686984	SAMN24003368	SRR17219706	OL842861	EPI_ISL_7332763
PRJNA686984	SAMN24003450	SRR17220331	OL842655	EPI_ISL_7332842
PRJNA686984	SAMN24297928	SRR17310815	OM005057	EPI_ISL_7830736
PRJNA686984	SAMN24297988	SRR17311230	OM005213	EPI_ISL_7830807
PRJNA686984	SAMN24297832	SRR17310850	OM005102	EPI_ISL_7830356
PRJNA686984	SAMN24298159	SRR17310968	OM005416	EPI_ISL_7830440
PRJNA686984	SAMN24298203	SRR17310956	OM004828	EPI_ISL_7830484
PRJNA686984	SAMN24297880	SRR17311098	OM004768	EPI_ISL_7830690
PRJNA686984	SAMN24297543	SRR17311291	OM005269	EPI_ISL_7830399
PRJNA686984	SAMN24297844	SRR17311281	OM004841	EPI_ISL_7830364
PRJNA686984	SAMN24297755	SRR17310755	OM005224	EPI_ISL_7830283
PRJNA686984	SAMN24297548	SRR17311271	OM005381	EPI_ISL_7830651
PRJNA686984	SAMN24297753	SRR17310758	OM004959	EPI_ISL_7830282
PRJNA686984	SAMN24298050	SRR17310788	OM004915	EPI_ISL_7830564
PRJNA686984	SAMN24297520	SRR17311019	OM005420	EPI_ISL_7830087
PRJNA686984	SAMN24297623	SRR17311383	OM004907	EPI_ISL_7830080
PRJNA686984	SAMN24297961	SRR17310707	OM005435	EPI_ISL_7830778
PRJNA686984	SAMN24297690	SRR17311129	OM005442	EPI_ISL_7830236
PRJNA686984	SAMN24297540	SRR17310735	OM004988	EPI_ISL_7830105
PRJNA686984	SAMN24297795	SRR17311300	OM005451	EPI_ISL_7830322
PRJNA686984	SAMN24297876	SRR17311102	OM005053	EPI_ISL_7830697
PRJNA686984	SAMN24297838	SRR17310843	OM005326	EPI_ISL_7830656
PRJNA686984	SAMN24297530	SRR17311327	OM004908	EPI_ISL_7830096
PRJNA686984	SAMN24298128	SRR17311002	OM005065	EPI_ISL_7830423
PRJNA686984	SAMN24297775	SRR17311322	OM005018	EPI_ISL_7830302
PRJNA686984	SAMN24297544	SRR17310870	OM004937	EPI_ISL_7830107

BioProject	BioSample	SRA	GenBank	GISAID
PRJNA686984	SAMN24297871	SRR17311252	OM005355	EPI_ISL_7830676
PRJNA686984	SAMN24297541	SRR17311313	OM004898	EPI_ISL_7830106
PRJNA686984	SAMN24298211	SRR17310947	OM004997	EPI_ISL_7830492
PRJNA686984	SAMN24297740	SRR17310880	OM005240	EPI_ISL_7830274
PRJNA686984	SAMN24297811	SRR17310874	OM005362	EPI_ISL_7830337
PRJNA686984	SAMN24297748	SRR17310763	OM004941	EPI_ISL_7830278
PRJNA686984	SAMN24297723	SRR17310899	OM004789	EPI_ISL_7830263
PRJNA686984	SAMN24297691	SRR17311128	OM004763	EPI_ISL_7830237
PRJNA686984	SAMN24297597	SRR17310667	OM005317	EPI_ISL_7830152
PRJNA686984	SAMN24297728	SRR17310893	OM004819	EPI_ISL_7830396
PRJNA686984	SAMN24390076	SRR17330212	OM015327	EPI_ISL_8064235
PRJNA686984	SAMN24389838	SRR17330261	OM015433	EPI_ISL_8063999
PRJNA686984	SAMN24389874	SRR17330893	OM015481	EPI_ISL_8064034
PRJNA686984	SAMN24389564	SRR17330412	OM015171	EPI_ISL_8063725
PRJNA686984	SAMN24389905	SRR17330858	OM015460	EPI_ISL_8064064
PRJNA686984	SAMN24389699	SRR17330487	OM014982	EPI_ISL_8063860
PRJNA686984	SAMN24389832	SRR17330376	OM014968	EPI_ISL_8063993
PRJNA686984	SAMN24389931	SRR17330830	OM015516	EPI_ISL_8064090
PRJNA686984	SAMN24389869	SRR17330226	OM015233	EPI_ISL_8064029
PRJNA686984	SAMN24389735	SRR17330411	OM015551	EPI_ISL_8063896
PRJNA686984	SAMN24390148	SRR17330660	OM015541	EPI_ISL_8064307
PRJNA686984	SAMN24389988	SRR17330802	OM015052	EPI_ISL_8064147
PRJNA686984	SAMN24390231	SRR17330569	OM015544	EPI_ISL_8064390
PRJNA686984	SAMN24389920	SRR17330842	OM015179	EPI_ISL_8064079
PRJNA686984	SAMN24389685	SRR17330502	OM015550	EPI_ISL_8063846
PRJNA686984	SAMN24389631	SRR17330528	OM015080	EPI_ISL_8063792
PRJNA686984	SAMN24389554	SRR17330524	OM014887	EPI_ISL_8063715
PRJNA686984	SAMN24389828	SRR17330380	OM014991	EPI_ISL_8063989
PRJNA686984	SAMN24389660	SRR17330914	OM015248	EPI_ISL_8063821
PRJNA686984	SAMN24389932	SRR17330829	OM015170	EPI_ISL_8064091
PRJNA686984	SAMN24390024	SRR17330763	OM015392	EPI_ISL_8064183
PRJNA686984	SAMN24390072	SRR17330217	OM015134	EPI_ISL_8064231
PRJNA686984	SAMN24390189	SRR17330615	OM015376	EPI_ISL_8064348
PRJNA686984	SAMN24390062	SRR17330720	OM015413	EPI_ISL_8064221
PRJNA686984	SAMN24389730	SRR17330417	OM015056	EPI_ISL_8063891
PRJNA686984	SAMN24389819	SRR17330390	OM015166	EPI_ISL_8063980
PRJNA686984	SAMN24389670	SRR17330903	OM014862	EPI_ISL_8063831
PRJNA686984	SAMN24390055	SRR17330727	OM015296	EPI_ISL_8064214
PRJNA686984	SAMN24390080	SRR17330208	OM015324	EPI_ISL_8064239
PRJNA686984	SAMN24470143	SRR17363145	OM063420	EPI_ISL_8065088
PRJNA686984	SAMN24470098	SRR17362766	OM063647	EPI_ISL_8065043
PRJNA686984	SAMN24470106	SRR17363079	OM063627	EPI_ISL_8065051
PRJNA686984	SAMN24469838	SRR17362755	OM063518	EPI_ISL_8064784
PRJNA686984	SAMN24470197	SRR17363046	OM063439	EPI_ISL_8065142
PRJNA686984	SAMN24469976	SRR17362967	OM063775	EPI_ISL_8064922
PRJNA686984	SAMN24469986	SRR17362956	OM063829	EPI_ISL_8064931
PRJNA686984	SAMN24469936	SRR17363303	OM063778	EPI_ISL_8064882
PRJNA686984	SAMN24469887	SRR17363357	OM063719	EPI_ISL_8064833
PRJNA686984	SAMN24470059	SRR17362912	OM063560	EPI_ISL_8065004
PRJNA686984	SAMN24470215	SRR17363026	OM063593	EPI_ISL_8065160
PRJNA686984	SAMN24470174	SRR17363254	OM063400	EPI_ISL_8065119
PRJNA686984	SAMN24470120	SRR17363064	OM063524	EPI_ISL_8065065
PRJNA686984	SAMN24470045	SRR17362927	OM063832	EPI_ISL_8064990
PRJNA686984	SAMN24470090	SRR17363168	OM063901	EPI_ISL_8065035
PRJNA686984	SAMN24470239	SRR17362892	OM063386	EPI_ISL_8065184
PRJNA686984	SAMN24470093	SRR17363164	OM063884	EPI_ISL_8065038
PRJNA686984	SAMN24469723	SRR17363246	OM063559	EPI_ISL_8064671
PRJNA686984	SAMN24470104	SRR17363081	OM063738	EPI_ISL_8065049
PRJNA686984	SAMN24470024	SRR17363205	OM063919	EPI_ISL_8064969
PRJNA686984	SAMN24470056	SRR17362915	OM063430	EPI_ISL_8065001
PRJNA686984	SAMN24470019	SRR17363211	OM063336	EPI_ISL_8064964
PRJNA686984	SAMN24469775	SRR17362825	OM063391	EPI_ISL_8064722
PRJNA686984	SAMN24591488	SRR17435033	OM169952	EPI_ISL_8159261
PRJNA686984	SAMN24591007	SRR17434824	OM169876	EPI_ISL_8158781
PRJNA686984	SAMN24591378	SRR17435049	OM170283	EPI_ISL_8159151
PRJNA686984	SAMN24591889	SRR17434657	OM169612	EPI_ISL_8159660
PRJNA686984	SAMN24591468	SRR17434173	OM170287	EPI_ISL_8159241
PRJNA686984	SAMN24591568	SRR17434344	OM169757	EPI_ISL_8159341
PRJNA686984	SAMN24591637	SRR17434972	OM169589	EPI_ISL_8159409

BioProject	BioSample	SRA	GenBank	GISAID
PRJNA686984	SAMN24591100	SRR17434476	OM169598	EPI_ISL_8158874
PRJNA686984	SAMN24757236	SRR17510699	OM191618	EPI_ISL_8336979
PRJNA686984	SAMN24757388	SRR17510383	OM191812	EPI_ISL_8337125
PRJNA686984	SAMN24757152	SRR17510321	OM191766	EPI_ISL_8336896
PRJNA686984	SAMN24757111	SRR17510547	OM191738	EPI_ISL_8336856
PRJNA686984	SAMN24757139	SRR17510335	OM191641	EPI_ISL_8336884
PRJNA686984	SAMN24757532	SRR17510620	OM191909	EPI_ISL_8337267
PRJNA686984	SAMN24757071	SRR17510172	OM191612	EPI_ISL_8336816
PRJNA686984	SAMN24757164	SRR17510308	OM191809	EPI_ISL_8336908
PRJNA686984	SAMN24757655	SRR17510519	OM191938	EPI_ISL_8337389
PRJNA686984	SAMN24757113	SRR17510545	OM191564	EPI_ISL_8336858
PRJNA686984	SAMN24757156	SRR17510316	OM192189	EPI_ISL_8336900
PRJNA686984	SAMN24757223	SRR17510715	OM191514	EPI_ISL_8336966
PRJNA686984	SAMN24757220	SRR17510718	OM191847	EPI_ISL_8336963
PRJNA686984	SAMN24757309	SRR17510435	Not submitted	EPI_ISL_8515047
PRJNA686984	SAMN24757394	SRR17510377	OM191924	EPI_ISL_8337131
PRJNA686984	SAMN24757618	SRR17510560	OM191810	EPI_ISL_8337352
PRJNA686984	SAMN24757679	SRR17510492	OM191995	EPI_ISL_8337413
PRJNA686984	SAMN24757267	SRR17510265	OM192103	EPI_ISL_8337009
PRJNA686984	SAMN24757447	SRR17510641	OM191674	EPI_ISL_8337184
PRJNA686984	SAMN24757130	SRR17510526	OM191711	EPI_ISL_8336875
PRJNA686984	SAMN24756990	SRR17510083	OM191565	EPI_ISL_8336736
PRJNA686984	SAMN24757467	SRR17510092	OM192098	EPI_ISL_8337204
PRJNA686984	SAMN24756937	SRR17510667	OM191667	EPI_ISL_8336683
PRJNA686984	SAMN24757355	SRR17510109	OM192187	EPI_ISL_8337092
PRJNA686984	SAMN24757622	SRR17510029	OM192020	EPI_ISL_8337356
PRJNA686984	SAMN24757566	SRR17510054	OM191807	EPI_ISL_8337302
PRJNA686984	SAMN24757123	SRR17510534	OM191885	EPI_ISL_8336868
PRJNA686984	SAMN24757251	SRR17510683	OM191749	EPI_ISL_8336994
PRJNA686984	SAMN24757250	SRR17510684	OM192171	EPI_ISL_8336993
PRJNA686984	SAMN24757249	SRR17510685	OM191588	EPI_ISL_8336992
PRJNA686984	SAMN24756954	SRR17510542	OM191825	EPI_ISL_8336700
PRJNA686984	SAMN24757143	SRR17510331	OM192174	EPI_ISL_8336888
PRJNA686984	SAMN24756966	SRR17510700	OM191962	EPI_ISL_8336712
PRJNA686984	SAMN24757088	SRR17510153	OM192018	EPI_ISL_8336833
PRJNA686984	SAMN24757090	SRR17510151	OM191872	EPI_ISL_8336835
PRJNA686984	SAMN24756975	SRR17510417	OM191630	EPI_ISL_8336721
PRJNA686984	SAMN24756969	SRR17510267	OM191866	EPI_ISL_8336715
PRJNA686984	SAMN24756998	SRR17510593	OM192178	EPI_ISL_8336744
PRJNA686984	SAMN24756938	SRR17510340	OM191839	EPI_ISL_8336684
PRJNA686984	SAMN24757558	SRR17510063	OM191601	EPI_ISL_8337294
PRJNA686984	SAMN24730737	SRR17497282	OM191209	EPI_ISL_8325978
PRJNA686984	SAMN24730427	SRR17497374	OM191367	EPI_ISL_8325672
PRJNA686984	SAMN24730214	SRR17497643	OM191444	EPI_ISL_8325460
PRJNA686984	SAMN24730281	SRR17497934	OM190857	EPI_ISL_8325526
PRJNA686984	SAMN24694615	SRR18294923	OM994161	EPI_ISL_8436508
PRJNA686984	SAMN24730820	SRR17497730	Not submitted	EPI_ISL_8326060
PRJNA686984	SAMN24694453	SRR18294980	OM994204	EPI_ISL_8436346
PRJNA686984	SAMN24730516	SRR17497986	OM190893	EPI_ISL_8325759
PRJNA686984	SAMN24730327	SRR17497593	OM191304	EPI_ISL_8325572
PRJNA686984	SAMN24730416	SRR17497638	OM190850	EPI_ISL_8325661
PRJNA686984	SAMN24730360	SRR17497557	OM191081	EPI_ISL_8325605
PRJNA686984	SAMN24730379	SRR17497536	OM191482	EPI_ISL_8325624
PRJNA686984	SAMN24730229	SRR17497333	OM191255	EPI_ISL_8325475
PRJNA686984	SAMN24730850	SRR17497445	Not submitted	EPI_ISL_8326089
PRJNA686984	SAMN24694450	SRR18294906	OM994208	EPI_ISL_8436343
PRJNA686984	SAMN24730905	SRR17497383	OM190969	EPI_ISL_8326144
PRJNA686984	SAMN24694532	SRR18294881	OM994215	EPI_ISL_8436425
PRJNA686984	SAMN24730790	SRR17497764	OM191387	EPI_ISL_8326030
PRJNA686984	SAMN24730927	SRR17497683	OM191161	EPI_ISL_8326166
PRJNA686984	SAMN24694609	SRR18294929	OM994240	EPI_ISL_8436502
PRJNA686984	SAMN24730768	SRR17497788	OM191391	EPI_ISL_8326009
PRJNA686984	SAMN24694577	SRR18294964	OM994222	EPI_ISL_8436470
PRJNA686984	SAMN24730817	SRR17497733	OM190935	EPI_ISL_8326057
PRJNA686984	SAMN24730743	SRR17497275	OM191196	EPI_ISL_8325984
PRJNA686984	SAMN24730535	SRR17497614	OM190790	EPI_ISL_8325778
PRJNA686984	SAMN24694582	SRR18294959	OM994243	EPI_ISL_8436475
PRJNA686984	SAMN24730799	SRR17497753	OM191151	EPI_ISL_8326039
PRJNA686984	SAMN24694519	SRR18294966	OM994106	EPI_ISL_8436412

BioProject	BioSample	SRA	GenBank	GISAID
PRJNA686984	SAMN24694524	SRR18294890	OM994140	EPI_ISL_8436417
PRJNA686984	SAMN24730247	SRR17497277	Not submitted	EPI_ISL_8325493
PRJNA686984	SAMN24730338	SRR17497581	OM191389	EPI_ISL_8325583
PRJNA686984	SAMN24730530	SRR17497620	OM191395	EPI_ISL_8325773
PRJNA686984	SAMN24694531	SRR18294882	OM994246	EPI_ISL_8436424
PRJNA686984	SAMN24694608	SRR18294930	OM994112	EPI_ISL_8436501
PRJNA686984	SAMN24694534	SRR18294879	OM994211	EPI_ISL_8436427
PRJNA686984	SAMN24730877	SRR17497415	OM190782	EPI_ISL_8326116
PRJNA686984	SAMN24694533	SRR18294880	OM994117	EPI_ISL_8436426
PRJNA686984	SAMN24730652	SRR17497844	OM191407	EPI_ISL_8325894
PRJNA686984	SAMN24730854	SRR17497440	OM191429	EPI_ISL_8326093
PRJNA686984	SAMN24965581	SRR17625366	OM282715	EPI_ISL_8564041
PRJNA686984	SAMN24730480	SRR17497496	OM191204	EPI_ISL_8325725
PRJNA686984	SAMN24730587	SRR17497980	OM190998	EPI_ISL_8325830
PRJNA686984	SAMN24694496	SRR18294992	OM994183	EPI_ISL_8436389
PRJNA686984	SAMN24730739	SRR17497280	OM190845	EPI_ISL_8325980
PRJNA686984	SAMN24730537	SRR17497612	OM191005	EPI_ISL_8325780
PRJNA686984	SAMN24730365	SRR17497551	OM191111	EPI_ISL_8325610
PRJNA686984	SAMN24730386	SRR17497528	OM190817	EPI_ISL_8325631
PRJNA686984	SAMN24694591	SRR18294949	OM994148	EPI_ISL_8436484
PRJNA686984	SAMN24730355	SRR17497562	OM191355	EPI_ISL_8325600
PRJNA686984	SAMN24730349	SRR17497569	OM191234	EPI_ISL_8325594
PRJNA686984	SAMN24694541	SRR18294871	OM994221	EPI_ISL_8436434
PRJNA686984	SAMN24694592	SRR18294948	OM994120	EPI_ISL_8436485
PRJNA686984	SAMN24694616	SRR18294922	OM994242	EPI_ISL_8436509
PRJNA686984	SAMN24694447	SRR18295036	OM994102	EPI_ISL_8436340
PRJNA686984	SAMN24694551	SRR18295029	OM994225	EPI_ISL_8436444
PRJNA686984	SAMN24694478	SRR18294904	OM994170	EPI_ISL_8436371
PRJNA686984	SAMN24694513	SRR18294973	OM994103	EPI_ISL_8436406
PRJNA686984	SAMN24694518	SRR18294967	OM994122	EPI_ISL_8436411
PRJNA686984	SAMN24694544	SRR18294868	OM994241	EPI_ISL_8436437
PRJNA686984	SAMN24965584	SRR17625362	OM282156	EPI_ISL_8564044
PRJNA686984	SAMN24694542	SRR18294870	OM994160	EPI_ISL_8436435
PRJNA686984	SAMN24694587	SRR18294953	OM994179	EPI_ISL_8436480
PRJNA686984	SAMN24965410	SRR17625103	OM282364	EPI_ISL_8563871
PRJNA686984	SAMN24694536	SRR18294877	OM994151	EPI_ISL_8436429
PRJNA686984	SAMN24694607	SRR18294931	OM994118	EPI_ISL_8436500
PRJNA686984	SAMN24694497	SRR18294990	OM994163	EPI_ISL_8436390
PRJNA686984	SAMN24694563	SRR18295016	OM994132	EPI_ISL_8436456
PRJNA686984	SAMN24694499	SRR18294988	OM994180	EPI_ISL_8436392
PRJNA686984	SAMN24809107	SRR17536227	OM213377	EPI_ISL_8516245
PRJNA686984	SAMN24908838	SRR17612340	OM268779	EPI_ISL_8643996
PRJNA686984	SAMN24809099	SRR17536236	OM213222	EPI_ISL_8516237
PRJNA686984	SAMN24808683	SRR17536590	OM213558	EPI_ISL_8515825
PRJNA686984	SAMN24809004	SRR17536269	OM213698	EPI_ISL_8516142
PRJNA686984	SAMN24808712	SRR17536378	OM213189	EPI_ISL_8515854
PRJNA686984	SAMN24808603	SRR17536426	OM213659	EPI_ISL_8515745
PRJNA686984	SAMN24808940	SRR17536666	OM213764	EPI_ISL_8516078
PRJNA686984	SAMN24808434	SRR17536482	OM213231	EPI_ISL_8515576
PRJNA686984	SAMN24809068	SRR17536487	OM213587	EPI_ISL_8516206
PRJNA686984	SAMN24808764	SRR17536113	OM213490	EPI_ISL_8515906
PRJNA686984	SAMN24808716	SRR17536373	OM213655	EPI_ISL_8515858
PRJNA686984	SAMN24808374	SRR17536464	OM213546	EPI_ISL_8515516
PRJNA686984	SAMN24808876	SRR17536061	OM213880	EPI_ISL_8516015
PRJNA686984	SAMN24808718	SRR17536371	OM213676	EPI_ISL_8515860
PRJNA686984	SAMN24808677	SRR17536597	OM213882	EPI_ISL_8515819
PRJNA686984	SAMN24808678	SRR17536596	OM213557	EPI_ISL_8515820
PRJNA686984	SAMN24808959	SRR17536537	OM213304	EPI_ISL_8516097
PRJNA686984	SAMN24808401	SRR17536344	OM213536	EPI_ISL_8515543
PRJNA686984	SAMN24808913	SRR17536020	OM213504	EPI_ISL_8516051
PRJNA686984	SAMN24808717	SRR17536372	OM213375	EPI_ISL_8515859
PRJNA686984	SAMN24808545	SRR17536141	OM213888	EPI_ISL_8515687
PRJNA686984	SAMN24808450	SRR17536208	OM213260	EPI_ISL_8515592
PRJNA686984	SAMN24808376	SRR17536441	OM213845	EPI_ISL_8515518
PRJNA686984	SAMN24808538	SRR17536149	OM213545	EPI_ISL_8515680
PRJNA686984	SAMN24808558	SRR17536127	OM213480	EPI_ISL_8515700
PRJNA686984	SAMN24808459	SRR17536198	OM213583	EPI_ISL_8515601
PRJNA686984	SAMN24808568	SRR17536758	OM213184	EPI_ISL_8515710
PRJNA686984	SAMN24808474	SRR17536462	OM213163	EPI_ISL_8515616

BioProject	BioSample	SRA	GenBank	GISAID
PRJNA686984	SAMN24808854	SRR17536086	OM213314	EPI_ISL_8515993
PRJNA686984	SAMN24808993	SRR17536281	OM213450	EPI_ISL_8516131
PRJNA686984	SAMN24808701	SRR17536390	OM213742	EPI_ISL_8515843
PRJNA686984	SAMN24808571	SRR17536755	OM213172	EPI_ISL_8515713
PRJNA686984	SAMN24808702	SRR17536389	OM213530	EPI_ISL_8515844
PRJNA686984	SAMN24808534	SRR17536153	OM213499	EPI_ISL_8515676
PRJNA686984	SAMN24808795	SRR17536574	OM213560	EPI_ISL_8515936
PRJNA686984	SAMN24808406	SRR17536577	Not submitted	EPI_ISL_8515548
PRJNA686984	SAMN24808402	SRR17536333	OM213452	EPI_ISL_8515544
PRJNA686984	SAMN24808975	SRR17536518	OM213409	EPI_ISL_8516113
PRJNA686984	SAMN24808833	SRR17536315	OM213378	EPI_ISL_8515972
PRJNA686984	SAMN24808682	SRR17536592	OM213303	EPI_ISL_8515824
PRJNA686984	SAMN24808519	SRR17536170	OM213311	EPI_ISL_8515661
PRJNA686984	SAMN24808681	SRR17536593	OM213332	EPI_ISL_8515823
PRJNA686984	SAMN24809005	SRR17536268	OM213457	EPI_ISL_8516143
PRJNA686984	SAMN24808493	SRR17536440	OM213802	EPI_ISL_8515635
PRJNA686984	SAMN24808553	SRR17536132	OM213874	EPI_ISL_8515695
PRJNA686984	SAMN24808578	SRR17536747	OM213593	EPI_ISL_8515720
PRJNA686984	SAMN24808579	SRR17536746	OM213739	EPI_ISL_8515721
PRJNA686984	SAMN24808582	SRR17536743	OM213481	EPI_ISL_8515724
PRJNA686984	SAMN24909338	SRR17611751	OM268766	EPI_ISL_8644495
PRJNA686984	SAMN24909343	SRR17611746	OM268368	EPI_ISL_8646904
PRJNA686984	SAMN24908691	SRR17612188	OM268297	EPI_ISL_8643849
PRJNA686984	SAMN24909313	SRR17611487	OM268223	EPI_ISL_8644470
PRJNA686984	SAMN24909000	SRR17612091	OM268424	EPI_ISL_8644158
PRJNA686984	SAMN24908573	SRR17612267	Not submitted	EPI_ISL_8643731
PRJNA686984	SAMN24909354	SRR17611980	OM268148	EPI_ISL_8644510
PRJNA686984	SAMN24851815	SRR17569880	OM241715	EPI_ISL_8594745
PRJNA686984	SAMN24851750	SRR17569736	OM241527	EPI_ISL_8594681
PRJNA686984	SAMN24851582	SRR17569565	OM241779	EPI_ISL_8594513
PRJNA686984	SAMN24909339	SRR17611750	OM268522	EPI_ISL_8644496
PRJNA686984	SAMN24909328	SRR17611762	OM268664	EPI_ISL_8644485
PRJNA686984	SAMN24908574	SRR17612256	Not submitted	EPI_ISL_8643732
PRJNA686984	SAMN24851559	SRR17569624	OM241714	EPI_ISL_8594490
PRJNA686984	SAMN24851771	SRR17569713	OM241853	EPI_ISL_8594701
PRJNA686984	SAMN24909207	SRR17611792	OM268744	EPI_ISL_8644365
PRJNA686984	SAMN24909494	SRR17611426	OM268256	EPI_ISL_8644648
PRJNA686984	SAMN24909386	SRR17611475	OM268557	EPI_ISL_8644542
PRJNA686984	SAMN24908843	SRR17612335	OM268178	EPI_ISL_8644001
PRJNA686984	SAMN24908686	SRR17612193	OM268592	EPI_ISL_8643844
PRJNA686984	SAMN24909209	SRR17611790	OM268257	EPI_ISL_8644367
PRJNA686984	SAMN24908931	SRR17611594	OM268537	EPI_ISL_8644089
PRJNA686984	SAMN24909210	SRR17611789	OM268532	EPI_ISL_8644368
PRJNA686984	SAMN24909391	SRR17611470	OM268288	EPI_ISL_8644547
PRJNA686984	SAMN24909100	SRR17612261	OM268200	EPI_ISL_8644258
PRJNA686984	SAMN24909238	SRR17612213	OM268309	EPI_ISL_8644396
PRJNA686984	SAMN24909020	SRR17612068	OM268473	EPI_ISL_8644178
PRJNA686984	SAMN24909282	SRR17611989	OM268363	EPI_ISL_8644440
PRJNA686984	SAMN24908865	SRR17612310	OM268198	EPI_ISL_8644023
PRJNA686984	SAMN24908929	SRR17611596	OM268369	EPI_ISL_8644087
PRJNA686984	SAMN24909158	SRR17611552	OM268345	EPI_ISL_8644316
PRJNA686984	SAMN24908681	SRR17612199	OM267985	EPI_ISL_8643839
PRJNA686984	SAMN24908968	SRR17612301	OM268339	EPI_ISL_8644126
PRJNA686984	SAMN24908995	SRR17612271	OM268785	EPI_ISL_8644153
PRJNA686984	SAMN24908994	SRR17612273	OM268412	EPI_ISL_8644152
PRJNA686984	SAMN24908893	SRR17612105	OM268646	EPI_ISL_8644051
PRJNA686984	SAMN24908894	SRR17612104	OM268362	EPI_ISL_8644052
PRJNA686984	SAMN24909211	SRR17611788	OM268336	EPI_ISL_8644369
PRJNA686984	SAMN25126843	SRR17680254	OM323343	EPI_ISL_8828913
PRJNA686984	SAMN25578831	SRR17854950	OM482246	EPI_ISL_9391358
PRJNA686984	SAMN25579246	SRR17855677	OM481936	EPI_ISL_9391772
PRJNA686984	SAMN25209255	SRR17714104	OM349321	EPI_ISL_9003481
PRJNA686984	SAMN26636359	SRR18545068	ON120747	EPI_ISL_10955710
PRJNA686984	SAMN26636490	SRR18544923	ON120715	EPI_ISL_10955838
PRJNA686984	SAMN25166012	SRR17701273	OM351833	EPI_ISL_8948928
PRJNA686984	SAMN25165502	SRR17701470	OM352007	EPI_ISL_8948420
PRJNA686984	SAMN26636376	SRR18545049	ON120589	EPI_ISL_10955727
PRJNA686984	SAMN26636214	SRR18545299	ON120688	EPI_ISL_10955568
PRJNA686984	SAMN25166041	SRR17700881	OM351988	EPI_ISL_8948956

BioProject	BioSample	SRA	GenBank	GISAID
PRJNA686984	SAMN26636198	SRR18545317	ON120619	EPI_ISL_10955552
PRJNA686984	SAMN26636494	SRR18544919	ON120786	EPI_ISL_10955842
PRJNA686984	SAMN25579146	SRR17855057	OM481868	EPI_ISL_9391673
PRJNA686984	SAMN25578782	SRR17855144	OM482332	EPI_ISL_9391309
PRJNA686984	SAMN25578790	SRR17855135	OM482320	EPI_ISL_9391317
PRJNA686984	SAMN25579454	SRR17855278	OM482294	EPI_ISL_9391976
PRJNA686984	SAMN25578974	SRR17855424	OM482210	EPI_ISL_9391501
PRJNA686984	SAMN25579252	SRR17855670	OM481816	EPI_ISL_9391778
PRJNA686984	SAMN25578851	SRR17855560	OM482349	EPI_ISL_9391378
PRJNA686984	SAMN25579227	SRR17855355	OM481967	EPI_ISL_9391753
PRJNA686984	SAMN25578824	SRR17854958	OM481899	EPI_ISL_9391351
PRJNA686984	SAMN25579361	SRR17855029	OM482111	EPI_ISL_9391886
PRJNA686984	SAMN25578757	SRR17855172	OM481621	EPI_ISL_9391284
PRJNA686984	SAMN25579042	SRR17854891	OM481760	EPI_ISL_9391569
PRJNA686984	SAMN25344822	SRR17797331	OM439209	EPI_ISL_9184082
PRJNA686984	SAMN25344657	SRR17797818	OM439514	EPI_ISL_9183919
PRJNA686984	SAMN25344442	SRR17797882	OM439532	EPI_ISL_9183704
PRJNA686984	SAMN25344741	SRR17797795	OM439174	EPI_ISL_9184003
PRJNA686984	SAMN25344748	SRR17797787	OM439266	EPI_ISL_9184010
PRJNA686984	SAMN25344604	SRR17797397	OM439570	EPI_ISL_9183866
PRJNA686984	SAMN25578779	SRR17855148	OM481913	EPI_ISL_9391306
PRJNA686984	SAMN25344445	SRR17797879	OM439417	EPI_ISL_9183707
PRJNA686984	SAMN25344360	SRR17797633	OM439044	EPI_ISL_9183622
PRJNA686984	SAMN25344413	SRR17797985	OM439492	EPI_ISL_9183675
PRJNA686984	SAMN25344498	SRR17797586	OM439539	EPI_ISL_9183760
PRJNA686984	SAMN25344757	SRR17797719	OM439330	EPI_ISL_9184019
PRJNA686984	SAMN25344777	SRR17797697	OM439068	EPI_ISL_9184038
PRJNA686984	SAMN25344826	SRR17797326	OM439459	EPI_ISL_9184085
PRJNA686984	SAMN25344752	SRR17797783	OM438994	EPI_ISL_9184014
PRJNA686984	SAMN25344673	SRR17797498	OM438900	EPI_ISL_9183935
PRJNA686984	SAMN25344436	SRR17797958	OM439281	EPI_ISL_9183698
PRJNA686984	SAMN25344795	SRR17797468	OM439290	EPI_ISL_9184056
PRJNA686984	SAMN25344694	SRR17797368	OM439231	EPI_ISL_9183956
PRJNA686984	SAMN25344762	SRR17797714	OM439344	EPI_ISL_9184024
PRJNA686984	SAMN25344542	SRR17797747	OM439264	EPI_ISL_9183804
PRJNA686984	SAMN25344666	SRR17797505	OM439213	EPI_ISL_9183928
PRJNA686984	SAMN25344534	SRR17797545	OM438968	EPI_ISL_9183796
PRJNA686984	SAMN25344805	SRR17797457	OM439095	EPI_ISL_9184066
PRJNA686984	SAMN25344554	SRR17797734	OM439398	EPI_ISL_9183816
PRJNA686984	SAMN25344421	SRR17797975	OM439355	EPI_ISL_9183683
PRJNA686984	SAMN25344755	SRR17797780	OM439490	EPI_ISL_9184017
PRJNA686984	SAMN25344788	SRR17797476	OM439139	EPI_ISL_9184049
PRJNA686984	SAMN25344609	SRR17797391	OM439126	EPI_ISL_9183871
PRJNA686984	SAMN25344607	SRR17797393	OM439374	EPI_ISL_9183869
PRJNA686984	SAMN25344751	SRR17797784	OM439561	EPI_ISL_9184013
PRJNA686984	SAMN25344596	SRR17797512	OM439259	EPI_ISL_9183858
PRJNA686984	SAMN25344653	SRR17797823	OM439376	EPI_ISL_9183915
PRJNA686984	SAMN25344800	SRR17797462	OM438925	EPI_ISL_9184061
PRJNA686984	SAMN25344446	SRR17797877	OM439291	EPI_ISL_9183708
PRJNA686984	SAMN25344548	SRR17797740	OM439060	EPI_ISL_9183810
PRJNA686984	SAMN25344520	SRR17797561	OM439180	EPI_ISL_9183782
PRJNA686984	SAMN25344444	SRR17797880	OM439295	EPI_ISL_9183706
PRJNA686984	SAMN25344539	SRR17797750	OM439147	EPI_ISL_9183801
PRJNA686984	SAMN25344759	SRR17797717	OM439300	EPI_ISL_9184021
PRJNA686984	SAMN25344821	SRR17797332	OM439177	EPI_ISL_9184081
PRJNA686984	SAMN25344612	SRR17797388	OM439541	EPI_ISL_9183874
PRJNA686984	SAMN25344573	SRR17797538	OM439141	EPI_ISL_9183835
PRJNA686984	SAMN25344572	SRR17797539	OM439548	EPI_ISL_9183834
PRJNA686984	SAMN25344775	SRR17797700	OM439464	EPI_ISL_9184036
PRJNA686984	SAMN25344546	SRR17797742	OM439552	EPI_ISL_9183808
PRJNA686984	SAMN25344608	SRR17797392	OM439326	EPI_ISL_9183870
PRJNA686984	SAMN25344537	SRR17797752	OM439381	EPI_ISL_9183799
PRJNA686984	SAMN25344606	SRR17797394	OM439237	EPI_ISL_9183868
PRJNA686984	SAMN25344581	SRR17797529	OM439235	EPI_ISL_9183843
PRJNA686984	SAMN25344747	SRR17797788	OM439367	EPI_ISL_9184009
PRJNA686984	SAMN25344834	SRR17797318	OM439455	EPI_ISL_9184093
PRJNA686984	SAMN25344686	SRR17797483	OM439472	EPI_ISL_9183948
PRJNA686984	SAMN25344602	SRR17797399	OM438891	EPI_ISL_9183864
PRJNA686984	SAMN25344626	SRR17797371	OM439383	EPI_ISL_9183888

BioProject	BioSample	SRA	GenBank	GISAID
PRJNA686984	SAMN25344586	SRR17797523	OM439280	EPI_ISL_9183848
PRJNA686984	SAMN25344355	SRR17797639	OM439439	EPI_ISL_9183617
PRJNA686984	SAMN25344545	SRR17797744	OM439473	EPI_ISL_9183807
PRJNA686984	SAMN25344670	SRR17797501	OM438926	EPI_ISL_9183932
PRJNA686984	SAMN25344528	SRR17797552	OM439394	EPI_ISL_9183790
PRJNA686984	SAMN25344544	SRR17797745	OM439268	EPI_ISL_9183806
PRJNA686984	SAMN25344414	SRR17797984	OM439238	EPI_ISL_9183676
PRJNA686984	SAMN25344487	SRR17797598	OM439431	EPI_ISL_9183749
PRJNA686984	SAMN25344418	SRR17797978	OM439444	EPI_ISL_9183680
PRJNA686984	SAMN25344587	SRR17797522	OM439191	EPI_ISL_9183849
PRJNA686984	SAMN25344425	SRR17797971	OM438945	EPI_ISL_9183687
PRJNA686984	SAMN25344753	SRR17797782	OM439489	EPI_ISL_9184015
PRJNA686984	SAMN25344553	SRR17797735	OM439182	EPI_ISL_9183815
PRJNA686984	SAMN25344350	SRR17797644	OM439294	EPI_ISL_9183612
PRJNA686984	SAMN25344522	SRR17797559	OM439240	EPI_ISL_9183784
PRJNA686984	SAMN25344597	SRR17797511	OM439239	EPI_ISL_9183859
PRJNA686984	SAMN25344531	SRR17797549	OM439419	EPI_ISL_9183793
PRJNA686984	SAMN25344639	SRR17797838	OM439304	EPI_ISL_9183901
PRJNA686984	SAMN25344423	SRR17797973	OM439350	EPI_ISL_9183685
PRJNA686984	SAMN25344802	SRR17797460	OM439554	EPI_ISL_9184063
PRJNA686984	SAMN25344781	SRR17797693	OM439388	EPI_ISL_9184042
PRJNA686984	SAMN25344754	SRR17797781	OM439197	EPI_ISL_9184016
PRJNA686984	SAMN25344691	SRR17797478	OM439093	EPI_ISL_9183953
PRJNA686984	SAMN25344388	SRR17797907	OM439056	EPI_ISL_9183650
PRJNA686984	SAMN25344427	SRR17797968	OM438912	EPI_ISL_9183689
PRJNA686984	SAMN25344525	SRR17797556	OM439311	EPI_ISL_9183787
PRJNA686984	SAMN25344582	SRR17797528	OM439037	EPI_ISL_9183844
PRJNA686984	SAMN25344399	SRR17797895	OM438885	EPI_ISL_9183661
PRJNA686984	SAMN25344550	SRR17797738	OM439254	EPI_ISL_9183812
PRJNA686984	SAMN25344379	SRR17797917	OM439146	EPI_ISL_9183641
PRJNA686984	SAMN25344536	SRR17797753	OM439418	EPI_ISL_9183798
PRJNA686984	SAMN25343561	SRR17797146	OM438319	EPI_ISL_9182773
PRJNA686984	SAMN25344541	SRR17797748	OM438924	EPI_ISL_9183803
PRJNA686984	SAMN25329775	SRR17791690	OM422569	EPI_ISL_9153683
PRJNA686984	SAMN25329758	SRR17791088	OM422587	EPI_ISL_9153666
PRJNA686984	SAMN25343808	SRR17797071	OM438720	EPI_ISL_9183019
PRJNA686984	SAMN25330119	SRR17791556	OM422128	EPI_ISL_9154027
PRJNA686984	SAMN25330297	SRR17791217	OM422250	EPI_ISL_9154200
PRJNA686984	SAMN25330036	SRR17791295	OM422190	EPI_ISL_9153944
PRJNA686984	SAMN26546052	SRR18300135	OM992920	EPI_ISL_10601154
PRJNA686984	SAMN26103479	SRR18082383	OM796622	EPI_ISL_10088665
PRJNA686984	SAMN26279698	SRR18155626	OM844578	EPI_ISL_10299985
PRJNA686984	SAMN26279587	SRR18155845	OM844443	EPI_ISL_10300145
PRJNA686984	SAMN26279586	SRR18155846	OM844674	EPI_ISL_10300144
PRJNA686984	SAMN26434319	SRR18229544	ON355698	EPI_ISL_10605449
PRJNA686984	SAMN26106121	SRR18084123	OM798525	EPI_ISL_10090818
PRJNA686984	SAMN26284872	SRR18157613	OM851609	EPI_ISL_10408978
PRJNA686984	SAMN26284894	SRR18157913	OM851233	EPI_ISL_10409000
PRJNA686984	SAMN26284788	SRR18157262	OM851623	EPI_ISL_10408895
PRJNA686984	SAMN26284559	SRR18157852	OM851524	EPI_ISL_10408667
PRJNA686984	SAMN26285106	SRR18157534	OM851562	EPI_ISL_10409212
PRJNA686984	SAMN26106175	SRR18084259	OM798084	EPI_ISL_10090872
PRJNA686984	SAMN26284527	SRR18157524	OM851099	EPI_ISL_10408635
PRJNA686984	SAMN26284682	SRR18157272	OM851549	EPI_ISL_10408789
PRJNA686984	SAMN26106172	SRR18084433	OM798406	EPI_ISL_10090869
PRJNA686984	SAMN26284925	SRR18157771	OM851326	EPI_ISL_10409031
PRJNA686984	SAMN26284910	SRR18157895	OM851452	EPI_ISL_10409016
PRJNA686984	SAMN26285027	SRR18157874	OM851564	EPI_ISL_10409133
PRJNA686984	SAMN26284928	SRR18157767	OM851147	EPI_ISL_10409034
PRJNA686984	SAMN26284507	SRR18157602	OM851098	EPI_ISL_10408615
PRJNA686984	SAMN26284878	SRR18157606	OM851597	EPI_ISL_10408984
PRJNA686984	SAMN26284499	SRR18157618	OM851110	EPI_ISL_10408607
PRJNA686984	SAMN26284848	SRR18157640	OM851641	EPI_ISL_10408954
PRJNA686984	SAMN26284856	SRR18157632	OM851658	EPI_ISL_10408962
PRJNA686984	SAMN26348200	SRR18188175	OM887178	EPI_ISL_10256989
PRJNA686984	SAMN26103093	SRR18081000	OM800205	EPI_ISL_10092002
PRJNA686984	SAMN26102941	SRR18081169	OM800457	EPI_ISL_10091850
PRJNA686984	SAMN26102800	SRR18081073	OM800789	EPI_ISL_10091709
PRJNA686984	SAMN26102877	SRR18081564	OM800700	EPI_ISL_10091786

BioProject	BioSample	SRA	GenBank	GISAID
PRJNA686984	SAMN26103041	SRR18081202	OM800502	EPI_ISL_10091950
PRJNA686984	SAMN26102782	SRR18081022	OM800419	EPI_ISL_10091691
PRJNA686984	SAMN26102875	SRR18081567	OM800275	EPI_ISL_10091784
PRJNA686984	SAMN26434410	SRR18583580	ON304655	EPI_ISL_10605541
PRJNA686984	SAMN26434288	SRR18583603	ON304664	EPI_ISL_10605417
PRJNA686984	SAMN26434724	SRR18583600	ON304675	EPI_ISL_10605860
PRJNA686984	SAMN26434716	SRR18583601	ON304614	EPI_ISL_10605852
PRJNA686984	SAMN26434418	SRR18583645	ON304676	EPI_ISL_10605549
PRJNA686984	SAMN26434694	SRR18583619	ON304632	EPI_ISL_10605830
PRJNA686984	SAMN26434453	SRR18583564	ON304683	EPI_ISL_10605585
PRJNA686984	SAMN26434295	SRR18583598	ON304630	EPI_ISL_10605424
PRJNA686984	SAMN26434443	SRR18583596	ON304633	EPI_ISL_10605574
PRJNA686984	SAMN26434605	SRR18583595	ON304663	EPI_ISL_10605739
PRJNA686984	SAMN26434528	SRR18583594	ON304657	EPI_ISL_10605661
PRJNA686984	SAMN26434840	SRR18583591	ON304669	EPI_ISL_10605978
PRJNA686984	SAMN26434476	SRR18583590	ON304646	EPI_ISL_10605608
PRJNA686984	SAMN26434294	SRR18583466	ON304690	EPI_ISL_10605423
PRJNA686984	SAMN26434602	SRR18583589	ON304689	EPI_ISL_10605736
PRJNA686984	SAMN26434400	SRR18583587	ON304618	EPI_ISL_10605531
PRJNA686984	SAMN26434276	SRR18583561	ON304613	EPI_ISL_10605405
PRJNA686984	SAMN26434702	SRR18583560	ON304641	EPI_ISL_10605838
PRJNA686984	SAMN26434286	SRR18583556	ON304621	EPI_ISL_10605415
PRJNA686984	SAMN26434616	SRR18583555	ON304626	EPI_ISL_10605751
PRJNA686984	SAMN26434460	SRR18583599	ON304682	EPI_ISL_10605592
PRJNA686984	SAMN26434838	SRR18583577	ON304661	EPI_ISL_10605976
PRJNA686984	SAMN26434450	SRR18583576	ON304600	EPI_ISL_10605582
PRJNA686984	SAMN26434824	SRR18583575	ON304648	EPI_ISL_10605962
PRJNA686984	SAMN26434888	SRR18583574	ON304615	EPI_ISL_10606025
PRJNA686984	SAMN26434274	SRR18583530	ON304692	EPI_ISL_10605403
PRJNA686984	SAMN26434469	SRR18583573	Not submitted	EPI_ISL_10605601
PRJNA686984	SAMN26434469	SRR18583573	Not submitted	EPI_ISL_10605601
PRJNA686984	SAMN26434665	SRR18583572	ON304624	EPI_ISL_10605800
PRJNA686984	SAMN26434401	SRR18583571	ON304666	EPI_ISL_10605532
PRJNA686984	SAMN26434406	SRR18583570	ON304625	EPI_ISL_10605537
PRJNA686984	SAMN26434814	SRR18583592	ON304612	EPI_ISL_10605952
PRJNA686984	SAMN26434739	SRR18583568	ON304642	EPI_ISL_10605876
PRJNA686984	SAMN26434578	SRR18583566	ON304616	EPI_ISL_10605712
PRJNA686984	SAMN26434776	SRR18583565	ON304599	EPI_ISL_10605913
PRJNA686984	SAMN26434890	SRR18583606	ON304684	EPI_ISL_10606027
PRJNA686984	SAMN26434709	SRR18583586	ON304651	EPI_ISL_10605845
PRJNA686984	SAMN26434296	SRR18583563	ON304605	EPI_ISL_10605425
PRJNA686984	SAMN26434407	SRR18583469	ON304698	EPI_ISL_10605538
PRJNA686984	SAMN26434384	SRR18583558	ON304634	EPI_ISL_10605515
PRJNA686984	SAMN26434512	SRR18583557	ON304610	EPI_ISL_10605645
PRJNA686984	SAMN26434474	SRR18583581	ON304623	EPI_ISL_10605606
PRJNA686984	SAMN26102933	SRR18081178	OM800563	EPI_ISL_10091842
PRJNA686984	SAMN26102929	SRR18081182	OM800627	EPI_ISL_10091838
PRJNA686984	SAMN26102955	SRR18081262	OM800565	EPI_ISL_10091864
PRJNA686984	SAMN26102835	SRR18081287	OM800583	EPI_ISL_10091744
PRJNA686984	SAMN26102892	SRR18081547	OM800422	EPI_ISL_10091801
PRJNA686984	SAMN26102893	SRR18081546	OM800338	EPI_ISL_10091802
PRJNA686984	SAMN26434805	SRR18583639	ON304693	EPI_ISL_10605943
PRJNA686984	SAMN26102986	SRR18081516	OM800351	EPI_ISL_10091895
PRJNA686984	SAMN26102954	SRR18081263	OM800512	EPI_ISL_10091863
PRJNA686984	SAMN26434765	SRR18583467	ON304679	EPI_ISL_10605902
PRJNA686984	SAMN26102972	SRR18081243	OM800196	EPI_ISL_10091881
PRJNA686984	SAMN26103129	SRR18081392	OM800321	EPI_ISL_10092038
PRJNA686984	SAMN26434564	SRR18583553	ON304656	EPI_ISL_10605698
PRJNA686984	SAMN26103114	SRR18081409	OM800725	EPI_ISL_10092023
PRJNA686984	SAMN26103038	SRR18081205	OM800795	EPI_ISL_10091947
PRJNA686984	SAMN26103103	SRR18080989	OM800486	EPI_ISL_10092012
PRJNA686984	SAMN26102987	SRR18081513	OM800708	EPI_ISL_10091896
PRJNA686984	SAMN26102850	SRR18081270	OM800633	EPI_ISL_10091759
PRJNA686984	SAMN26102883	SRR18081558	OM800542	EPI_ISL_10091792
PRJNA686984	SAMN26102830	SRR18081292	OM800699	EPI_ISL_10091739
PRJNA686984	SAMN26102944	SRR18081166	OM800596	EPI_ISL_10091853
PRJNA686984	SAMN26102796	SRR18081078	OM800553	EPI_ISL_10091705
PRJNA686984	SAMN26102862	SRR18081581	OM800497	EPI_ISL_10091771
PRJNA686984	SAMN26102898	SRR18081540	OM800374	EPI_ISL_10091807

BioProject	BioSample	SRA	GenBank	GISAID
PRJNA686984	SAMN26103030	SRR18081214	OM800204	EPI_ISL_10091939
PRJNA686984	SAMN26102976	SRR18081239	OM800288	EPI_ISL_10091885
PRJNA686984	SAMN26102805	SRR18081068	OM800818	EPI_ISL_10091714
PRJNA686984	SAMN26102845	SRR18081276	OM800656	EPI_ISL_10091754
PRJNA686984	SAMN26102912	SRR18081525	OM800362	EPI_ISL_10091821
PRJNA686984	SAMN26102931	SRR18081180	OM800741	EPI_ISL_10091840
PRJNA686984	SAMN26275125	SRR18153895	OM855273	EPI_ISL_10253511
PRJNA686984	SAMN26348084	SRR18188520	OM887359	EPI_ISL_10256873
PRJNA686984	SAMN26348517	SRR18188363	OM886923	EPI_ISL_10257305
PRJNA686984	SAMN26348500	SRR18188382	OM887160	EPI_ISL_10257288
PRJNA686984	SAMN26348208	SRR18188165	OM887006	EPI_ISL_10256997
PRJNA686984	SAMN26275089	SRR18153792	OM854907	EPI_ISL_10253475
PRJNA686984	SAMN26275040	SRR18154014	OM855185	EPI_ISL_10253425
PRJNA686984	SAMN26275022	SRR18154034	OM855136	EPI_ISL_10253406
PRJNA686984	SAMN26274953	SRR18153853	OM855104	EPI_ISL_10253335
PRJNA686984	SAMN26274920	SRR18153751	OM855299	EPI_ISL_10253302
PRJNA686984	SAMN26275464	SRR18154192	OM855006	EPI_ISL_10253853
PRJNA686984	SAMN26275018	SRR18154039	OM854883	EPI_ISL_10253402
PRJNA686984	SAMN26275552	SRR18154346	OM854938	EPI_ISL_10253944
PRJNA686984	SAMN26275455	SRR18154202	OM855531	EPI_ISL_10253844
PRJNA686984	SAMN26275201	SRR18153738	OM855402	EPI_ISL_10253589
PRJNA686984	SAMN26275050	SRR18154003	Not submitted	EPI_ISL_10253436
PRJNA686984	SAMN26275104	SRR18153774	OM855181	EPI_ISL_10253489
PRJNA686984	SAMN26275078	SRR18153804	OM854924	EPI_ISL_10253464
PRJNA686984	SAMN26275483	SRR18154171	OM855288	EPI_ISL_10253873
PRJNA686984	SAMN26275333	SRR18154265	OM855133	EPI_ISL_10253721
PRJNA686984	SAMN26275037	SRR18154018	OM855077	EPI_ISL_10253422
PRJNA686984	SAMN26288197	SRR18160456	OM859657	EPI_ISL_10410831
PRJNA686984	SAMN26434396	SRR18229622	ON355861	EPI_ISL_10605527
PRJNA686984	SAMN26587630	SRR18311947	OM997633	EPI_ISL_10954633
PRJNA686984	SAMN26587288	SRR18312039	OM997555	EPI_ISL_10954293
PRJNA686984	SAMN26587611	SRR18312148	OM997512	EPI_ISL_10954614
PRJNA686984	SAMN26587421	SRR18311999	OM998074	EPI_ISL_10954426
PRJNA686984	SAMN26587390	SRR18312214	OM997608	EPI_ISL_10954395
PRJNA686984	SAMN26587370	SRR18312236	OM998051	EPI_ISL_10954375
PRJNA686984	SAMN26945808	SRR18484805	ON078298	EPI_ISL_11103755
PRJNA686984	SAMN27060310	SRR18525837	ON102421	EPI_ISL_11435444
PRJNA686984	SAMN27278305	SRR18589237	ON201549	EPI_ISL_11627109

Appendix Table 2. Descriptive characteristics of 1,177 children with positive SARS-CoV-2 tests, Colorado, January 2021-January 2022 that were successfully sequenced and received a lineage call*

		Lineage group			p
Characteristic	Total	Other	Delta	Omicron	value†
Total population	1,177	189	654	334	-
Sex					
F	379 (32.2) [29.5–34.9]	88 (46.7) [39.3–53.9]	246 (37.6) [33.9–41.5]	81 (24.2) [19.8–29.1]	0.94
M	431 (36.6) [33.9–39.4]	97 (51.3) [44.0–58.6]	286 (43.7) [39.9–47.6]	90 (26.9) [22.3–32.1]	
Median age, y (range)	5.7 (0–20.9)	10.6 (0–20.2)	6.1 (0–20.1)	2.2 (0–20.9)	-
Racial-ethnic group					
Hispanic All Races	235 (19.9) [17.7–22.4]	54 (28.6) [22.3–35.6]	159 (24.3) [21.1–27.8]	49 (14.7) [11.1–18.9]	-
Non-Hispanic White	296 (25.2) [22.7–27.7]	87 (46.0) [38.8–53.4]	205 (31.3) [27.8–35.1]	36 (10.8) [7.7–14.6]	<0.0001
Non-Hispanic Black	61 (5.2) [4.0–6.6]	13 (6.9) [3.7–11.5]	33 (5.0) [3.5–7.0]	20 (6.0) [3.7–9.1]	
Non-Hispanic Asian	24 (2.0) [1.1–3.0]	8 (4.2) [1.8–8.2]	12 (1.8) [1.0–3.2]	6 (1.8) [0.7–3.9]	
Unknown‡	420 (35.7) [32.9–38.5]	12 (6.3) [3.3–10.3]	204 (31.2) [27.7–34.9]	209 (63.6) [57.1–67.8]	
Hospitalization status					
Outpatient	1,053 (89.5) [87.6–91.2]	148 (78.3) [71.7–84.0]	586 (89.6) [87.0–91.8]	260 (78.1) [73.0–82.2]	<0.0001
Inpatient	183 (15.5) [13.5–17.8]	41 (21.7) [16.0–28.3]	68 (10.4) [8.2–13.0]	74 (22.2) [17.8–27.0]	
Any comorbidity (inpatient only)§	114 (62.3) [54.9–69.3]	29 (70.7) [54.5–83.9]	42 (61.8) [49.2–73.3]	43 (58.2) [46.1–69.5]	0.41
Median time from symptom onset to testing, d (range)	2 (–2 to 320)	2 (0–19)	2 (–2 to 320)	2 (–2 to 15)	-

*Values are no. (%) [95% CI] except as indicated.
†P values were obtained from χ^2 test of patient characteristics across the 3 variant periods.
‡Unknown racial-ethnic group includes individuals with unknown, unknown or not reported, or missing race or ethnicity variables. NH = Non-Hispanic.
§Any comorbidity includes past medical history of cardiac, respiratory, gastrointestinal/liver, neurologic, oncologic, obesity, chronic kidney disease, diabetes, or other comorbid condition.

Appendix Table 3. Descriptive characteristics of 2,299 children with positive SARS-CoV-2 tests, Colorado, January 2021–January 2022 based on whether Ct values were able to be collected*

Characteristic	Total	Ct collected	Ct not collected	p value†
Total population	2299	1796	503	-
Sex				
F	923 (40.1) [38.1–42.2]	677 (37.7) [35.5–40.0]	247 (49.1) [44.7–53.6]	0.81
M	945 (41.1) [39.1–43.2]	697 (38.8) [36.6–41.1]	248 (49.3) [44.9–53.8]	
Median age, y (range)	6.5 [0–21]	6.8 [0–21]	5.9 [0–19.8]	-
Racial-ethnic group				
Hispanic All Races	560 (24.4) [22.6–26.2]	405 (22.6) [20.6–24.6]	155 (30.8) [26.8–35.1]	-
Non-Hispanic White	750 (32.6) [30.7–34.6]	527 (29.3) [27.2–31.5]	223 (44.7) [39.9–48.8]	<0.0001
Non-Hispanic Black	135 (5.9) [5.0–6.9]	103 (5.7) [4.7–6.9]	32 (6.4) [4.4–8.9]	
Non-Hispanic Asian	43 (1.9) [1.4–2.5]	32 (1.8) [1.2–2.5]	11 (2.2) [1.1–3.9]	
Unknown‡	673 (29.3) [27.4–31.2]	450 (25.2) [23.1–27.1]	53 (10.5) [8.0–13.6]	
Hospitalization status				
Outpatient	1629 (70.9) [69.0–72.7]	1434 (79.8) [77.9–81.7]	195 (38.8) [34.5–43.2]	<0.0001
Inpatient	670 (29.1) [27.3–31.1]	362 (20.2) [18.3–22.1]	308 (61.2) [56.9–65.5]	
Any comorbidity§ (inpatient only)	383 (57.2) [53.3–61.0]	219 (60.5) [55.5–65.5]	183 (59.4) [53.7–65.0]	0.06
Median time from symptom onset to testing, d (range)	2 (–29 to 320)	2 (–2 to 320)	2 (–29 to 85)	-

*Values are no. (%) [95% CI] except as indicated. Ct, cycle threshold.
†P values were obtained from χ^2 test of patient characteristics on the basis of whether a Ct value was collected.
‡Unknown racial-ethnic group includes individuals with unknown, unknown or not reported, or missing race or ethnicity variables.
§Any comorbidity includes past medical history of cardiac, respiratory, gastrointestinal/liver, neurologic, oncologic, obesity, chronic kidney disease, diabetes, or other comorbid condition.

Appendix Table 4. Proportions of inpatients by lineage group and potential indicator of disease severity, Colorado, January 2021-January 2022 for samples that were successfully sequenced and received a lineage call

Indicator of disease severity*	No. (%) [95% CI]				p value†
	Total (n = 112)	Other (n = 21)	Delta (n = 41)	Omicron (n = 50)	
Symptomatic	111 (99.1) [95.1–100]	21 (100) [83.9–100]	40 (97.6) [87.1–99.9]	50 (100) [92.9–100]	0.02
Hospitalized	112 (100) [96.8–100]	21 (100) [83.9–100]	41 (100) [91.4–100]	50 (100) [92.9–100]	1.0
PICU admitted	35 (31.1) [22.8–40.7]	7 (33.3) [14.6–57.0]	17 (41.4) [26.3–57.9]	11 (22.0) [11.5–36.0]	0.11
Received any oxygen support	75 (67.0) [57.4–75.6]	14 (66.7) [43.0–85.4]	29 (70.7) [54.5–83.9]	32 (64.0) [49.2–77.1]	0.79

*All indicators of disease severity included here were considered to be because of COVID-19 illness.
†P values were obtained from χ^2 test of patient characteristics across the 3 variant periods.

Appendix Table 5. Proportions of inpatients by lineage group and potential indicator of disease severity, Colorado, January 2021-January 2022 separated by whether Ct values were able to be collected.

Indicators of disease severity*	No. (%) [95% CI]			p value†
	Total (n = 395)	Ct collected (n = 184)	Ct not collected (n = 181)	
Symptomatic	343 (86.8) [83.1–90.0]	172 (93.5) [88.9–96.6]	171 (94.5) [90.1–97.3]	0.57
Hospitalized	395 (100) [99.1–100]	184 (100) [98.0–100]	181 (100) [98.0–100]	1.0
PICU Admitted	94 (23.8) [19.7–28.3]	58 (31.5) [24.9–38.8]	36 (19.9) [14.3–26.5]	0.12
Received any oxygen support	217 (54.9) [49.9–59.9]	113 (61.4) [54.0–68.5]	104 (57.5) [49.9–64.8]	0.51

*All indicators of disease severity included here were considered to be because of COVID-19 illness.
†P values were obtained from χ^2 test of patient characteristics on the basis of whether a Ct value was collected.

Appendix Table 6. Statistical analysis of N gene Ct values are response variables and predictor variables of time frame, race/ethnicity group, age bin, sex, days between symptom onset and test (onset_days_bin), patient type (includes outpatient, inpatient with COVID-19, and inpatient due to COVID-19), vaccination status, symptoms, hospital stay duration, comorbidity, PICU admission, and receipt of oxygen support*

Analysis	Df	Sum Sq	Mean Sq	F value	Pr(>F)	Significance code†
Analysis of Covariance (ANCOVA):						
Response N gene Ct:						
age_bin	3	312.9	104.304	1.3534	0.25669	
age_bin:onset_days_bin	8	804	100.496	1.304	0.23972	
age_bin:race_ethnicity_group	15	1132	75.465	0.9792	0.47638	
age_bin:symptomatic	3	152.5	50.818	0.6594	0.57746	
age_bin:time_frame	6	414.7	69.11	0.905	0.491514	
age_bin:vax_any	2	81.6	40.809	0.5295	0.58931	
onset_days_bin	3	562.3	187.447	2.4322	0.06465	.
onset_days_bin:race_ethnicity_group	13	924.3	71.096	0.9225	0.52947	
onset_days_bin:time_frame	6	1490.1	248.35	3.2523	0.004113	**
onset_days_bin:vax_any	3	382.2	127.398	1.653	0.17665	
patient_type	2	114.3	57.149	0.7415	0.47704	
patient_type:age_bin	6	472	78.667	1.0207	0.41118	
patient_type:onset_days_bin	6	657.7	109.61	1.4222	0.20466	
patient_type:sex	2	126.2	63.108	0.8189	0.44168	
patient_type:race_ethnicity_group	9	642.4	71.374	0.9261	0.50214	
patient_type:vax_any	2	109.7	54.849	0.7117	0.49144	
patient_type:time_frame	4	223.2	55.79	0.7306	0.571639	
sex	1	44.3	44.252	0.5742	0.44905	
sex:age_bin	3	163.8	54.601	0.7085	0.54739	
sex:onset_days_bin	3	267.7	89.247	1.158	0.32556	
sex:race_ethnicity_group	5	55.8	11.155	0.1447	0.98152	
sex:symptomatic	1	148.1	148.104	1.9217	0.16645	
sex:time_frame	2	307.1	153.53	2.0106	0.135712	
sex:vax_any	1	21.8	21.752	0.2822	0.59553	
race_ethnicity_group	5	70.8	14.168	0.1838	0.96864	
race_ethnicity_group:time_frame	10	562.1	56.21	0.7361	0.690231	
symptomatic	1	4.3	4.26	0.0553	0.81425	
symptomatic:race_ethnicity_group	4	591.7	147.928	1.9194	0.10639	
symptomatic:time_frame	2	12	6.02	0.0788	0.924244	
symptomatic:vax_any	1	179.5	179.45	2.3284	0.12783	
time_frame	2	248.7	124.37	1.6286	0.197928	
vax_any	1	0.5	0.513	0.0067	0.93501	
vax_any:race_ethnicity_group	5	323.9	64.771	0.8404	0.52158	
vax_any:time_frame	2	179.9	89.95	1.1779	0.309332	
Response N gene Ct (inpatient only)‡:	Df	Sum Sq	Mean Sq	F value	Pr(>F)	Significance code
any_comorbidity	1	21.7	21.748	0.3045	0.58193	
any_comorbidity:picu_due_to_covid	1	0.1	0.09	0.0013	0.97168	
any_comorbidity:respiratory_support_any	1	27	26.993	0.3779	0.53968	
hospital_duration_bins	2	44.8	22.405	0.3137	0.73125	
hospital_duration_bins:any_comorbidity	2	357.6	178.785	2.503	0.08534	.
hospital_duration_bins:picu_due_to_covid	1	20.3	20.26	0.2837	0.59513	
hospital_duration_bins:respiratory_support_any	1	17.4	17.423	0.2439	0.62213	
picu_due_to_covid	1	0	0.001	0	0.99724	
picu_due_to_covid:respiratory_support_any	1	57.6	57.621	0.8067	0.37057	
respiratory_support_any	1	3.3	3.323	0.0465	0.82953	
time_frame	2	16.6	8.294	0.1161	0.89045	
time_frame:any_comorbidity	2	1.8	0.922	0.0129	0.98718	
time_frame:hospital_duration_bins	4	308.7	77.183	1.0806	0.36836	
time_frame:picu_due_to_covid	2	150.3	75.166	1.0523	0.35175	
time_frame:respiratory_support_any	2	0.6	0.28	0.0039	0.99608	
Analysis of Variance (ANOVA)§:						
Response N gene Ct:	Df	Sum Sq	Mean Sq	F value	Pr(>F)	Significance code
time_frame (one-way)	2	916	458	9.972	6.30E-05	***
age_bin	3	1591	530.3	11.662	1.60E-07	***
age_bin:time_frame	6	770	128.3	2.822	0.00994	**
vax_any	1	260	260.2	5.749	0.0166	*
time_frame:vax_any	2	62	31	0.685	0.5043	
symptomatic	1	96	96.14	2.098	0.1478	
symptomatic:time_frame	2	208	103.87	2.266	0.1042	
onset_days_bin	2	1284	641.9	16.582	9.85E-08	***
onset_days_bin:time_frame	4	256	64.1	1.656	0.159	
patient_type	1	54	54.09	1.248	0.2641	
patient_type:time_frame	2	163	81.53	1.881	0.1529	

Analysis	Df	Sum Sq	Mean Sq	F value	Pr(>F)	Significance code†
Response N gene Ct (inpatient only):	Df	Sum Sq	Mean Sq	F value	Pr(>F)	Significance code
picu_due_to_covid	1	17	17.06	0.42	0.5179	
picu_due_to_covid:time_frame	2	53	26.42	0.65	0.5233	
respiratory_support_any	1	558	557.7	13.028	0.000371	***
respiratory_support_any:time_frame	2	274	136.8	3.196	0.042622	*
hospital_duration_bins	2	45	22.4	0.323	0.724	
time_frame:hospital_duration_bins	4	306	76.53	1.104	0.356	
any_comorbidity	1	10	10.1	0.144	0.704	
time_frame:any_comorbidity	2	16	7.85	0.112	0.894	

*Predictor variables and covariates are separated by a colon. While all possible pairwise comparisons were performed within data categories across variant periods, comparisons considered in this study include those between categories but within variant period (e.g., age groups within Delta) and the same category across variant periods (e.g., 1–4 y olds across pre-Delta, Delta, and Omicron). As such, comparisons not relevant to the current study were not included in post-hoc test†† (e.g., 12+ years during Omicron vs. <1 y during Delta). Ct, cycle threshold; N, nucleocapsid; PICU, pediatric intensive care unit.

†Significance codes: 0.001 '***', 0.01 '**', 0.05 '*', 0.1 '.', 1 ' '.

‡Inpatient because of COVID-19.

§Two-way ANOVA unless otherwise noted, ANOVAs were followed up with post-hoc Tukey HSD, p values from post-hoc tests are presented in the figures and text.

Appendix Table 7. Statistical analysis of N gene Ct values are response variables and predictor variables of sequenced lineage, race/ethnicity group, age bin, sex, days between symptom onset and test (onset_days_bin), patient type (includes outpatient, inpatient with COVID-19, and inpatient due to COVID-19), vaccination status, symptoms, hospital stay duration, comorbidity, PICU admission, and receipt of oxygen support*

Analysis	Df	Sum Sq	Mean Sq	F value	Pr(>F)	Significance code†
Analysis of Covariance (ANCOVA)						
Response N gene Ct						
age_bin	3	223.7	74.573	4.3261	0.005572	**
age_bin:lineage	6	35.5	5.911	0.3429	0.913406	
age_bin:onset_days_bin	6	44	7.329	0.4252	0.861676	
age_bin:race_ethnicity_group	15	428.6	28.572	1.6575	0.062045	.
age_bin:symptomatic	3	22.2	7.385	0.4284	0.732864	
age_bin:vax_any	2	11.1	5.57	0.3231	0.724247	
lineage	2	551.7	275.848	16.0023	3.58E-07	***
onset_days_bin	3	134.6	44.881	2.6036	0.053081	.
onset_days_bin:lineage	4	55.8	13.957	0.8096	0.520317	
onset_days_bin:race_ethnicity_group	7	71.1	10.156	0.5892	0.764341	
onset_days_bin:vax_any	3	26.1	8.705	0.505	0.679257	
patient_type	2	52.7	26.339	1.5279	0.219505	
patient_type:age_bin	6	134.3	22.39	1.2988	0.25926	
patient_type:lineage	4	92.5	23.126	1.3416	0.255831	
patient_type:onset_days_bin	5	131.2	26.231	1.5217	0.184563	
patient_type:sex	2	15.7	7.865	0.4562	0.634319	
patient_type:race_ethnicity_group	8	156.8	19.603	1.1372	0.339838	
patient_type:vax_any	2	33.1	16.572	0.9614	0.384126	
sex	1	124.2	124.175	7.2036	0.007886	**
sex:age_bin	3	29.1	9.702	0.5628	0.640131	
sex:lineage	2	6.4	3.192	0.1852	0.8311	
sex:onset_days_bin	3	80.2	26.726	1.5504	0.202738	
sex:race_ethnicity_group	5	140.2	28.041	1.6267	0.154491	
sex:symptomatic	1	1.8	1.783	0.1035	0.748058	
sex:vax_any	1	2.5	2.494	0.1447	0.704102	
race_ethnicity_group	5	29.2	5.831	0.3383	0.889311	
race_ethnicity_group:lineage	10	112.9	11.285	0.6547	0.765354	
symptomatic	1	28.2	28.224	1.6373	0.202175	
symptomatic:lineage	2	69	34.52	2.0025	0.137692	
symptomatic:race_ethnicity_group	4	72.2	18.045	1.0468	0.384177	
symptomatic:vax_any	1	49.7	49.749	2.886	0.090907	.
vax_any	1	9.1	9.145	0.5305	0.467244	
vax_any:lineage	2	25.8	12.923	0.7497	0.473847	
vax_any:race_ethnicity_group	5	110.7	22.145	1.2846	0.271914	
Response N gene Ct (inpatient only)‡	Df	Sum Sq	Mean Sq	F value	Pr(>F)	Significance code
any_comorbidity	1	69.23	69.226	5.7846	0.0181	*
any_comorbidity:picu_due_to_covid	1	12.37	12.367	1.0334	0.31193	
any_comorbidity:respiratory_support_any	1	8.56	8.556	0.715	0.39992	
hospital_duration_bins	2	1.99	0.996	0.0832	0.9202	
hospital_duration_bins:any_comorbidity	1	45.65	45.653	3.8148	0.05374	.
hospital_duration_bins:picu_due_to_covid	1	13.32	13.322	1.1132	0.29405	

Analysis	Df	Sum Sq	Mean Sq	F value	Pr(>F)	Significance code†
hospital_duration_bins:respiratory_support_any	1	0.88	0.876	0.0732	0.78729	
lineage	2	70.32	35.16	2.9381	0.0578	.
lineage:any_comorbidity	2	6.29	3.147	0.263	0.7693	
lineage:hospital_duration_bins	3	77.93	25.977	2.1707	0.09655	.
lineage:picu_due_to_covid	2	8.77	4.383	0.3662	0.69432	
lineage:respiratory_support_any	2	45.11	22.555	1.8848	0.1575	
picu_due_to_covid	1	7.48	7.475	0.6246	0.4313	
picu_due_to_covid:respiratory_support_any	1	4.59	4.593	0.3838	0.53705	
respiratory_support_any	1	4.48	4.482	0.3745	0.542	
Analysis of Variance (ANOVA)§						
Response N gene Ct:	Df	Sum Sq	Mean Sq	F value	Pr(>F)	Significance code
lineage (one-way)	2	703	351.5	20.87	1.27E-09	***
age_bin	3	430	143.3	8.672	1.14E-05	***
age_bin:lineage	6	194	32.3	1.952	0.0701	.
vax_any	1	29	28.5	1.698	0.193	
lineage:vax_any	2	11	5.6	0.334	0.716	
symptomatic	1	6	6.46	0.374	0.541	
symptomatic:lineage	2	30	15.05	0.87	0.419	
onset_days_bin	2	349	174.36	11.28	1.64E-05	***
onset_days_bin:lineage	4	67	16.87	1.092	0.36	
patient_type	1	2	1.54	0.093	0.76	
patient_type:lineage	2	6	2.81	0.17	0.844	
Response N gene Ct (inpatient only):	Df	Sum Sq	Mean Sq	F value	Pr(>F)	Significance code*
picu_due_to_covid	1	8	8	0.553	0.459	
picu_due_to_covid:lineage	2	13.5	6.76	0.467	0.628	
respiratory_support_any	1	0.4	0.45	0.03	0.86381	
respiratory_support_any:lineage	2	53.1	26.55	1.755	0.17629	
hospital_duration_bins	2	2	1	0.08	0.9231	
lineage:hospital_duration_bins	3	80.8	26.95	2.168	0.0957	.
any_comorbidity	1	62.5	62.48	5.046	0.0266	*
lineage:any_comorbidity	2	1.3	0.64	0.051	0.95	

* Predictor variables and covariates are separated by a colon. While all possible pairwise comparisons were performed within data categories across lineage groups, comparisons considered in this study include those between categories but within lineage group (e.g., age groups within Delta) and the same category across variant periods (e.g., 1–4 y olds across Other, Delta, and Omicron). As such, comparisons not relevant to the current study were not included in post-hoc test[††] (e.g., 12+ years during Omicron vs. <1 y during Delta). Ct, cycle threshold; N, nucleocapsid; PICU, pediatric intensive care unit.

†Significance codes: 0.001 '***', 0.01 '**', 0.05 '*', 0.1 '.', 1 ' '.

‡Inpatient DUE to COVID-19

§Two-way ANOVA unless otherwise noted, ANOVAs were followed up with post-hoc Tukey HSD, p values from post-hoc tests are presented in the figures and text.

Appendix Table 8. Mean nucleocapsid gene cycle threshold values (±SD) by category

Category		All	Other	Delta	Omicron
All	-	18.9 (4.17)	18.9 (4.36)	18.3 (4.29)	20.1 (3.59)
Age bin	<1	17.3 (4.25)	18.2 (4.69)	16.0 (4.19)	19.0 (3.43)
	1–4 y	19.1 (4.08)	20.0 (3.28)	18.2 (4.36)	20.2 (3.18)
	5–11 y	19.0 (4.10)	18.5 (4.28)	18.9 (4.06)	20.5 (3.81)
	≥12 years	19.1 (4.34)	18.7 (4.58)	18.3 (4.13)	21.9 (3.19)
Symptom onset bins	0–3 d	18.1 (4.09)	18.3 (4.12)	17.4 (4.12)	19.5 (3.63)
	4–7 d	18.9 (3.80)	17.3 (2.27)	18.7 (4.05)	18.7 (3.42)
	8–14 d	21.5 (3.72)	22.7 (2.38)	20.9 (3.67)	24.1 (4.24)
Vaccination status	Unvaccinated	18.9 (4.17)	18.5 (4.36)	18.2 (4.30)	20.1 (3.61)
	Vaccinated (any doses)	19.3 (4.19)	19.6 (4.31)	18.7 (4.24)	20.7 (3.38)
	Partially vaccinated (1 dose)	18.3 (4.37)	20.4 (0.93)	17.2 (4.29)	22.1 (4.13)
	Fully vaccinated (2 doses)	19.4 (4.18)	19.3 (4.33)	19.1 (4.26)	20.6 (3.42)
	fully vaccinated + booster	20.0 (4.00)	20.3 (4.71)	18.8 (1.91)	19.8 (2.75)
Disease severity	Outpatient	18.9 (4.17)	19.0 (4.36)	18.3 (4.29)	20.1 (3.54)
	Inpatient (hospitalized with COVID-19)	19.5 (5.12)	18.4 (5.45)	17.5 (5.06)	21.6 (4.29)
	Inpatient (hospitalized due to COVID-19)	18.8 (3.82)	18.3 (3.66)	18.2 (4.18)	19.7 (3.39)
	Symptomatic	18.6 (4.16)	18.5 (4.12)	18.1 (4.27)	19.9 (3.63)
	Asymptomatic	18.8 (4.30)	19.3 (4.68)	18.0 (4.30)	20.4 (3.42)
	Admitted to PICU due to COVID-19	18.4 (3.37)	19.0 (3.99)	17.9 (3.90)	18.9 (1.91)
	Hospitalized but not admitted to PICU	19.0 (3.98)	18.0 (3.6)	18.4 (4.34)	20.0 (3.66)
	No supplemental oxygen	18.9 (3.97)	17.4 (2.26)	17.4 (4.48)	20.7 (3.45)
	Any supplemental oxygen	18.8 (4.08)	17.8 (5.07)	18.6 (3.99)	19.4 (3.65)
	Noninvasive supplemental oxygen	19.1 (3.97)	18.8 (4.57)	18.9 (4.05)	19.3 (3.73)
	Invasive supplemental oxygen	16.1 (4.28)	11.1 (3.36)	17.0 (3.53)	20.4 (NA)

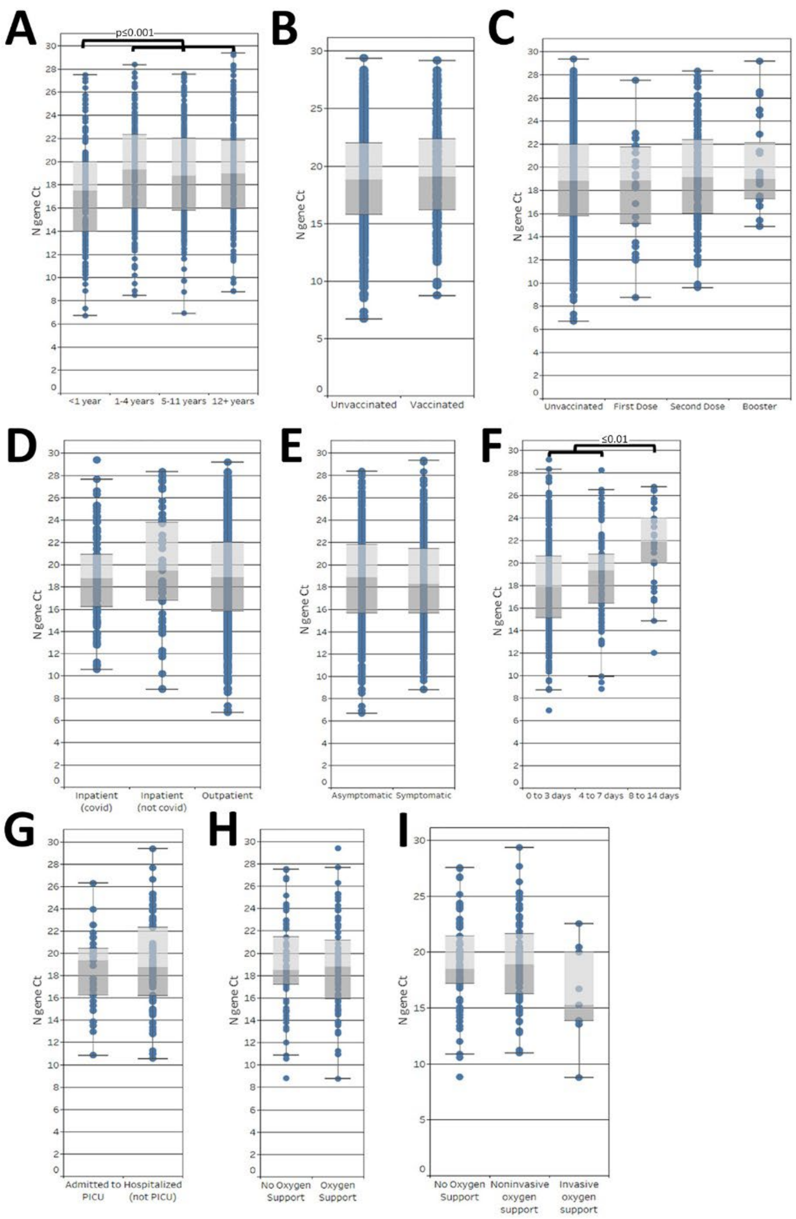

Appendix Figure 1. Ct value patterns across all variants among successfully sequenced samples. Boxplots of overall Ct value patterns across categories among samples that were successfully sequenced. Ct values by A) age group, B) vaccination status (unvaccinated vs. vaccinated with any number of doses), C) vaccination status (unvaccinated vs. vaccinated by number of doses), D) patient type (outpatient/not hospitalized, hospitalized due to COVID-19, hospitalized but not due to COVID-19), E) symptomatic vs. asymptomatic, F) number of days between symptom onset and positive test (symptom onset group), G) hospitalized but not admitted to PICU vs. admitted to PICU, H) any type of supplemental oxygen support vs. no oxygen support received, I) highest level of supplemental oxygen support received (none, noninvasive oxygen support, invasive oxygen support). n.s., not significant, (*), adjusted $p \leq 0.05$, (**) adjusted $p \leq 0.01$.

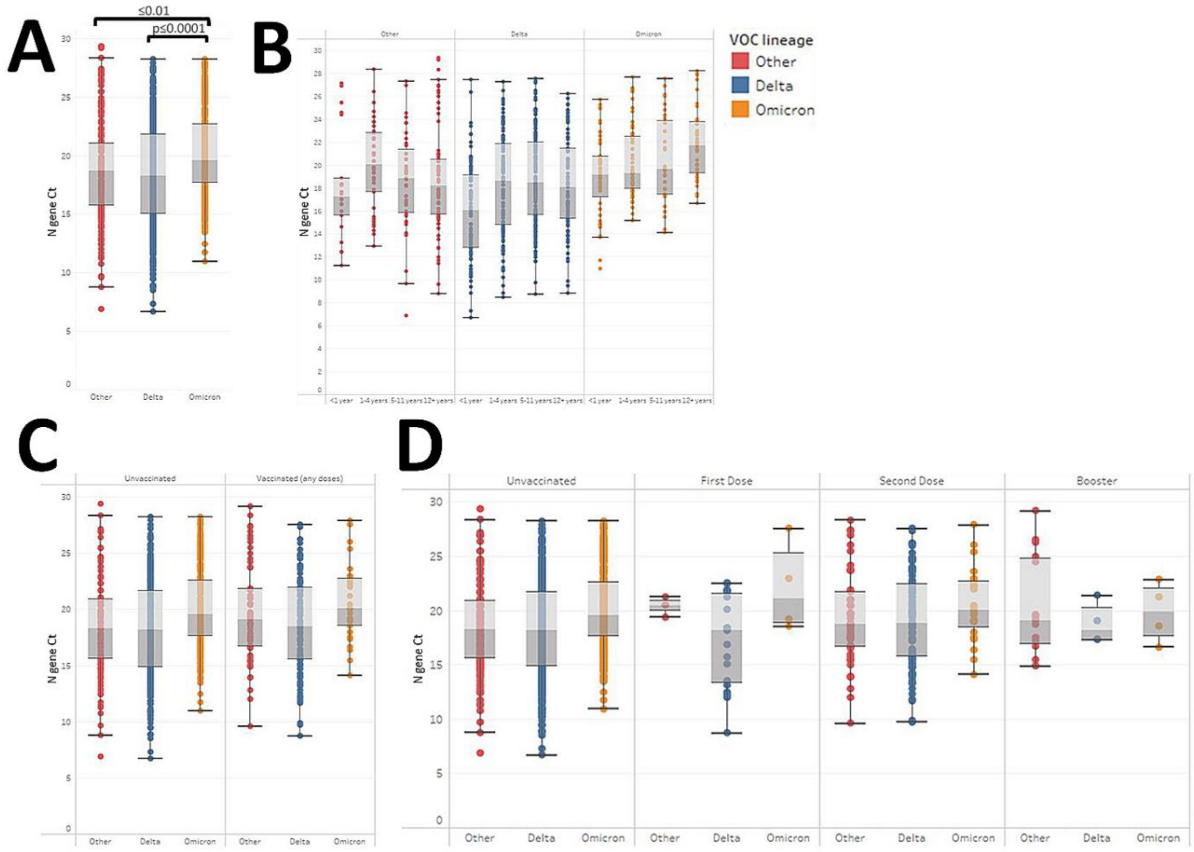

Appendix Figure 2. Ct value patterns of patient characteristics among successfully sequenced samples. Boxplots of overall Ct value patterns across categories among successfully sequenced samples. Ct values by A) variant period, B) age groups, C) vaccination status (unvaccinated vs. vaccinated with any number of doses), D) vaccination status (unvaccinated vs. vaccinated by number of doses). (*), adjusted p≤0.05; (**) adjusted p≤0.01, (***) adjusted p≤0.001; (****) adjusted p≤0.0001.

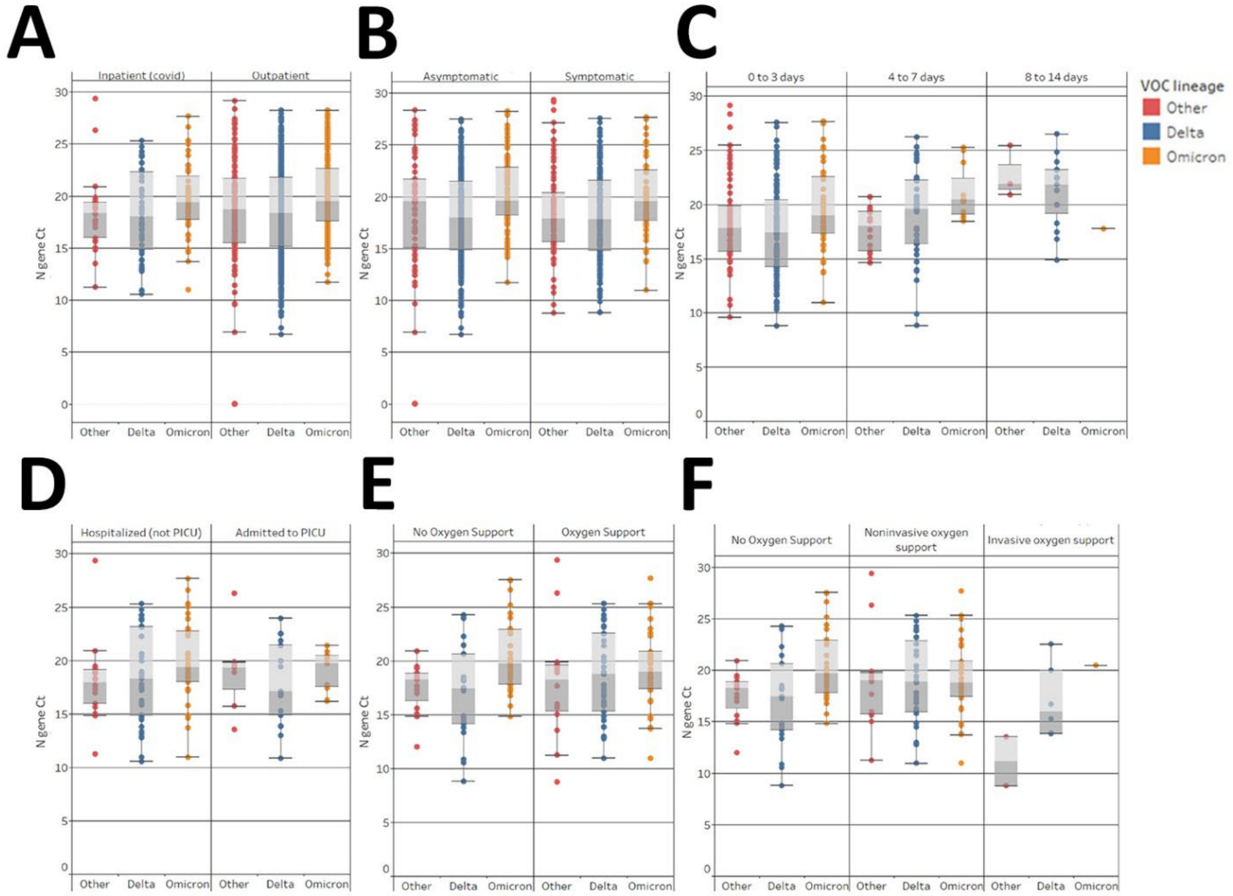

Appendix Figure 3. Ct value patterns among markers of disease severity among successfully sequenced samples. Boxplots of overall Ct value patterns across disease severity markers among successfully sequenced samples. Ct values by A) patient type (outpatient/not hospitalized, hospitalized due to COVID-19), B) symptomatic vs. asymptomatic, C) number of days between symptom onset and positive test (symptom onset group), D) hospitalized but not admitted to PICU vs. admitted to PICU, E) any type of supplemental oxygen support vs. no oxygen support received, F) highest level of supplemental oxygen support received (none, noninvasive oxygen support, invasive oxygen support). (*), adjusted p≤0.05; (**) adjusted p≤0.01, (***) adjusted p≤0.001; (****) adjusted p≤0.0001.
